# Transcriptomic Analysis of Potential “lncRNA–mRNA” Interactions in Liver of the Marine Teleost *Cynoglossus semilaevis* Fed Diets With Different DHA/EPA Ratios

**DOI:** 10.3389/fphys.2019.00331

**Published:** 2019-04-02

**Authors:** Houguo Xu, Lin Cao, Bo Sun, Yuliang Wei, Mengqing Liang

**Affiliations:** ^1^Yellow Sea Fisheries Research Institute, Chinese Academy of Fishery Sciences, Qingdao, China; ^2^Laboratory for Marine Fisheries Science and Food Production Processes, Qingdao National Laboratory for Marine Science and Technology, Qingdao, China; ^3^Beijing Institute of Feed Control, Beijing, China; ^4^Wuxi Fisheries College, Nanjing Agricultural University, Wuxi, China

**Keywords:** *Cynoglossus semilaevis*, diet, DHA/EPA, hepatic transcriptome, lncRNA

## Abstract

Long non-coding RNAs (lncRNA) have emerged as important regulators of lipid metabolism and have been shown to play multifaceted roles in controlling transcriptional gene regulation, but very little relevant information has been available in fish, especially in non-model fish species. With a feeding trial on a typical marine teleost tongue sole *C. semilaevis* followed by transcriptomic analysis, the present study investigated the possible involvement of lncRNA in hepatic mRNA expression in response to different levels of dietary DHA and EPA, which are two most important fatty acids for marine fish. An 80-day feeding trial was conducted in a flow-through seawater system, and in this trial three experimental diets differing basically in DHA/EPA ratio, i.e., 0.61 (D/E-0.61), 1.46 (D/E-1.46), and 2.75 (D/E-2.75), were randomly assigned to 9 tanks of experimental fish. A total of 124.04 G high quality genome-wide clean data about coding and non-coding transcripts was obtained in the analysis of hepatic transcriptome. Compared to diet D/E-0.61, D/E-1.46 up-regulated expression of 178 lncRNAs and 2629 mRNAs, and down-regulated that of 47 lncRNAs and 3059 mRNAs, while D/E-2.75 resulted in much less change in gene expression. The co-expression and co-localization analysis of differentially expressed (DE) lncRNA and mRNA among dietary groups were then conducted. The co-expressed DE lncRNA and mRNA were primarily enriched in GO terms such as Metabolic process, Intracellular organelle, Catalytic activity, and Oxidoreductase activity, as well as in KEGG pathways such as Ribosome and Oxidative phosphorylation. Overlap of co-expression and co-localization analysis, i.e., lncRNA–mRNA matches “XR_523541.1–solute carrier family 16, member 5 (*slc16a5*)” and “LNC_000285–bromodomain adjacent to zinc finger domain 2A (*baz2a*),” were observed in all inter-group comparisons, indicating that they might crucially mediate the effects of dietary DHA and EPA on hepatic gene expression in tongue sole. In conclusion, this was the first time in marine teleost to investigate the possible lncRNA–mRNA interactions in response to dietary fatty acids. The results provided novel knowledge of lncRNAs in non-model marine teleost, and will serve as important resources for future studies that further investigate the roles of lncRNAs in lipid metabolism of marine teleost.

## Introduction

Long non-coding RNAs (lncRNAs) are a class of RNA molecules with more than 200 bases that function as RNAs with little or no protein-coding capacity (Spizzo et al., 2012). LncRNAs represent a new frontier in molecular biology. More and more studies have demonstrated that lncRNAs play critical roles in various biological processes, including chromatin modification, regulation of transcription, influence of nuclear architecture and regulation of gene expression at post-transcriptional and post-translational levels, and also interact with DNA, RNA and proteins during these processes (Zhu et al., 2013; Kornfeld and Brüning, 2014; Gardini and Shiekhattar, 2015; Lopez-Pajares, 2016; Smekalova et al., 2016; Delás and Hannon, 2017; Long et al., 2017).

LncRNAs have emerged as important regulators of lipid metabolism, and have been shown to influence lipid homeostasis by controlling lipid metabolism in the liver and by regulating adipogenesis (Chen, 2016). In humans and other mammals, loss- and gain-of-function analysis of identified lncRNAs showed that lncRNAs are important regulators of a series of lipid metabolism-related processes such as plasma triglyceride accumulation (Cui et al., 2015; Li et al., 2015), cholesterol transportation, apolipoprotein A1 (APOA1) expression (Halley et al., 2014; Hu et al., 2014), adipose proliferation (Xu et al., 2010; Liu et al., 2014a,b), brown adipose tissue activation (Alvarez-Dominguez et al., 2015), and adipogenesis (Cooper et al., 2014; Divoux et al., 2014; Zhao et al., 2014; Gernapudi et al., 2015; Xiao et al., 2015).

In fish, however, little information has been available either about the identification and characterization of lncRNAs or about the roles of lncRNAs in lipid metabolism. Limited studies on lncRNAs in fish have been restricted to model fish species, and most of the studies focused on developmental biology (Kaushik et al., 2013; Liu et al., 2013; Haque et al., 2014; Dhiman et al., 2015; Al-Tobasei et al., 2016; Wang et al., 2016, 2017; Sarangdhar et al., 2017; Hu et al., 2018). Therefore, following our previous studies on lipid/fatty acid nutrition of marine fish (Zuo et al., 2012a,b; Xu et al., 2016, 2017), the present study was aimed at investigating the potential involvement of lncRNAs in effects of dietary fatty acids on a non-model marine teleost tongue sole *Cynoglossus semilaevis*, which is also an important aquaculture species. A feeding trial was conducted in this study, followed by hepatic transcriptome analysis.

Docosahexaenoic acid (DHA, 22:6n-3) and eicosapentaenoic acid (EPA, 20:5n-3) are the most important essential fatty acids for marine fish. Previous studies have widely demonstrated the critical roles of DHA and EPA in a series of physiological functions of marine fish such as functions of visual and neural systems (Bell et al., 1995; Furuita and Takeuchi, 1998; Ishizaki et al., 2000, 2001; Benítez-Santana et al., 2007; Noffs et al., 2009), bone development (Gapasin and Duray, 2001; Roo et al., 2009), pigmentation (Villalta et al., 2008; Vizcaíno-Ochoa et al., 2010), stress resistance (Kanazawa, 1997; Liu et al., 2002), immune response (Zuo et al., 2012b; Xu et al., 2016), and reproduction (Wilson, 2009; Xu et al., 2017). However, more fundamental mechanisms involved are still elusive. How DHA and EPA regulate physiological processes at transcriptional and post-transcriptional levels needs to be elucidated. Moreover, compared to total DHA+EPA contents, the effects of DHA/EPA ratio were interesting as well but relatively less studied (Sargent et al., 1999; Kim et al., 2002; Lee et al., 2003; Wu et al., 2003; Skalli and Robin, 2004; Hamre and Harboe, 2008; Wilson, 2009; Lund and Steenfeldt, 2011; ØStbye et al., 2011; Tocher, 2015). Our previous studies with marine species such as large yellow croaker *Larmichthys crocea* (Zuo et al., 2012a), Japanese seabass *Lateolabrax japonicus* (Xu et al., 2016) and tongue sole (Xu et al., 2017) have shown the significantly different roles of DHA and EPA in some physiological processes such as non-specific immune response and gonadal steroidogenesis. In the present study, taking advantage of completely sequenced genome data on tongue sole *C. semilaevis*, a genome-wide hepatic transcriptome analysis was conducted with tongue sole following an 80-day feeding trial with diets containing different DHA/EPA ratios. Potential lncRNA–mRNA interactions were thereafter analyzed based on the co-expression and co-localization analysis of differentially expressed lncRNAs and mRNAs among dietary groups. The results will provide novel knowledge about lncRNAs in non-model marine fish, and will serve as important resources for future studies that further investigate the roles of lncRNAs in lipid metabolism of marine teleost.

## Materials and Methods

### Experimental Diets, Experimental Fish, and Feeding Procedure

The experimental diets, experimental fish and feeding procedure has been described in a previous study of ours (Xu et al., 2018). Briefly, different levels of EPA enriched oil (containing 11.2% DHA and 52.0% EPA; in the form of triglyceride; Xi'an Renbang Biological Science and Technology Co., Ltd., Xi'an, China) and DHA enriched oil (containing 69.5% DHA and 6.6% EPA; in the form of triglyceride; Xi'an Renbang Biological Science and Technology Co., Ltd., Xi'an, China) were supplemented to the basal diet to obtain different DHA/EPA ratios, 0.61, 1.46, and 2.75, and the corresponding diets were designated as D/E-0.61, D/E-1.46, and D/E-2.75, respectively (Tables 1,2).

**Table 1:** Formulation and proximate composition of the experiment diets (g kg^−1^ dry matter).

**Ingredient**	**D/E-0.61**	**D/E-1.46**	**D/E-2.75**
Fish meal	400.0	400.0	400.0
Soybean meal	200.0	200.0	200.0
Wheat gluten	120.0	120.0	120.0
Wheat meal	144.0	144.0	144.0
Vitamin premix^a^	10.0	10.0	10.0
Mineral premix^b^	10.0	10.0	10.0
Monocalcium phosphate	10.0	10.0	10.0
Choline chloride	10.0	10.0	10.0
L-ascorbyl-2-polyphosphate	2.0	2.0	2.0
Ethoxyquin	0.5	0.5	0.5
Soy lecithin	20.0	20.0	20.0
Soybean oil	15.0	15.0	15.0
ARA enriched oil^c^	6.0	6.0	6.0
Olive oil	18.6	21.2	22.7
EPA enriched oil^d^	29.9	14.0	5.0
DHA enriched oil^e^	4.0	17.3	24.8
**PROXIMATE COMPOSITION**			
Crude protein	531.1	532.2	533.7
Crude lipid	118.2	119.2	121.6
Ash	114.1	114.6	114.4

a *Vitamin premix (mg or g/kg diet): thiamin 25 mg; riboflavin, 45 mg; pyridoxine HCl, 20 mg; vitamin B_12_, 0.1 mg; vitamin K_3_, 10 mg; inositol, 800 mg; pantothenic acid, 60 mg; niacin, 200 mg; folic acid, 20 mg; biotin, 1.2 mg; retinol acetate, 32 mg; cholecalciferol, 5 mg; alpha-tocopherol, 120 mg; wheat middling, 13.67 g*.

b *Mineral premix (mg or g/kg diet): MgSO_4_·7H_2_O, 1200 mg; CuSO_4_·5H_2_O, 10 mg; ZnSO_4_·H_2_O, 50 mg; FeSO_4_·H_2_O, 80 mg; MnSO_4_·H_2_O, 45 mg; CoCl_2_·6H_2_O (1%), 50 mg; NaSeSO_3_·5H_2_O (1%), 20mg; Ca(IO_3_)_2_·6H_2_O (1%), 60mg; zoelite, 13.485 g*.

c *ARA enriched oil: containing 41.0% ARA (of total fatty acids); in the form of triglyceride; Jiangsu Tiankai Biotechnology Co., Ltd., Nanjing, China*.

d *EPA enriched oil: containing 11.2% DHA and 52.0% EPA (of total fatty acids); in the form of triglyceride; Xi'an Renbang Biological Science and Technology Co., Ltd., Xi'an, China*.

e *DHA enriched oil: containing 69.5% DHA and 6.6% EPA (of total fatty acids); in the form of triglyceride; Xi'an Renbang Biological Science and Technology Co., Ltd., Xi'an, China*.

**Table 2:** Fatty acid compositions of the experimental diets (% total fatty acids).

**Fatty acid**	**D/E-0.61**	**D/E-1.46**	**D/E-2.75**
C14:0	1.36	1.35	1.36
C16:0	13.68	14.02	13.96
C18:0	3.88	3.77	3.67
∑SFA	18.93	19.15	18.98
C16:1n-7	1.52	1.56	1.64
C18:1n-9	20.72	21.79	21.83
C18:1n-7	2.14	1.99	1.81
∑MUFA	24.37	25.35	25.27
C18:2n-6	21.18	21.19	21.15
C20:4n-6	3.69	3.15	2.91
∑n-6 PUFA	24.87	24.34	24.07
C18:3n-3	2.27	2.22	2.24
C20:5n-3	13.92	8.94	5.93
C22:5n-3	1.36	2.03	2.43
C22:6n-3	8.54	13.06	16.32
∑n-3 PUFA	26.10	26.25	26.92
∑n-3 LC-PUFA	23.83	24.03	24.68
∑n-3/∑n-6	1.05	1.08	1.12
DHA/EPA	0.61	1.46	2.75

*SFA, saturated fatty acid; MUFA, monounsaturated fatty acid; PUFA, polyunsaturated fatty acid; LC-PUFA, long chain-polyunsaturated fatty acid*.

The feeding trial was conducted in a flow-through seawater system. Juvenile tongue sole *C. semilaevis* with an average initial weight of 23.40 ± 0.45 g were used in the feeding trial. Each diet was randomly assigned to triplicate tanks of 30 fish each. Fish were hand-fed to apparent satiation twice daily. The feeding trial lasted for 80 days. At the end of the feeding trial, after anesthetized with eugenol, liver samples from 6 fish each tank were collected, frozen with liquid nitrogen and stored at −80°C prior to analysis. The feeding trial was carried out in accordance with the recommendations of Guidelines on Management of Experimental Animals, Animal Care and Use Committee of Yellow Sea Fisheries Research Institute. All sampling protocols, as well as fish rearing practices, were reviewed and approved by the Animal Care and Use Committee of Yellow Sea Fisheries Research Institute.

### RNA Isolation, cDNA Library Construction, and Illumina Sequencing

Total RNA in liver samples was isolated using RNAiso Plus (TaKaRa Biotechnology (Dalian) Co., Ltd., Dalian, China). RNA degradation and contamination were monitored on 1% agarose gels. No genomic DNA contamination was observed. RNA purity was checked using the Nano Photometer® spectrophotometer (IMPLEN, Westlake Village, CA, USA). RNA concentration was measured using Qubit® RNA Assay Kit in Qubit® 2.0 Flurometer (Life Technologies, Carlsbad, CA, USA). RNA integrity was assessed using the RNA Nano 6000 Assay Kit of the Bioanalyzer 2100 system (Agilent Technologies, Santa Clara, CA, USA).

A total amount of 3 μg RNA per sample was used as input material for the RNA sample preparations and all samples had RIN values > 8. Six individual samples from the same experimental tank were pooled in equal amounts to obtain a pooling sample for this replicate tank. Nine pooling samples were then used to prepare 9 separate Illumina sequencing libraries (three biological replicates for each dietary group).

After ribosomal RNA was removed with Epicentre Ribo-Zero^TM^ rRNA Removal Kit (Epicentre, Madison, WI, USA), and rRNA free residues were cleaned up by ethanol precipitation, sequencing libraries were generated using the rRNA-depleted RNA by NEBNext® Ultra™ Directional RNA Library Prep Kit for Illumina® (NEB, USA) following the manufacturer's instructions. Briefly, fragmentation was carried out using divalent cations under elevated temperature in NEBNext First Strand Synthesis Reaction Buffer (5×). First strand cDNA was synthesized using random hexamer primer and M-MuLV Reverse Transcriptase (RNase H^−^). Second strand cDNA synthesis was subsequently performed using DNA Polymerase I and RNase H. Remaining overhangs were converted into blunt ends via exonuclease/polymerase activities. After adenylation of 3′ ends of DNA fragments, NEBNext Adaptor with hairpin loop structure was ligated to prepare for hybridization. In order to select cDNA fragments of 150 ~200 bp in length, the library fragments were purified with AMPure XP system (Beckman Coulter, Beverly, MA, USA). Then 3 μl USER Enzyme (NEB, Ipswich, MA, USA) was added to size-selected and adaptor-ligated cDNA at 37°C for 15 min followed by 5 min of 95°C treatment before the PCR process. PCR was then carried out with Phusion High-Fidelity DNA polymerase, Universal PCR primers and Index (X) Primer. At last, PCR products were purified with AMPure XP system (Beckman Coulter, Beverly, USA), and the library quality was assessed on Agilent Bioanalyzer 2100 system (Agilent, Santa Clara, USA).

The clustering of the index-coded samples was performed on a cBot Cluster Generation System using TruSeq PE Cluster Kit v3-cBot-HS (Illumina) according to the manufacturer's instructions. After cluster generation, the libraries were sequenced on an Illumina Hiseq2500 platform (Illumina, Inc., San Diego, CA, USA) and 150 bp paired-end reads were generated.

Raw data (raw reads) in fastq format were firstly processed through in-house perl scripts. Clean data (clean reads) were obtained by removing reads containing adapter or poly-N, as well as low quality reads from raw data. At the same time, Q20, Q30, and GC content of the clean data were calculated. All the downstream analysis were based on clean data with high quality. The paired-end clean reads were then aligned to the reference genome (RefSeq assembly accession: GCA_000523025.1; https://www.ncbi.nlm.nih.gov/genome/?term=Cynoglossus%20semilaevis) using TopHat v2.0.9. The mapped reads of each sample were assembled by both Scripture (beta2) (Guttman et al., 2010) and Cufflinks (v2.1.1) (Trapnell et al., 2010) in a reference-based approach. Cuffdiff (v2.1.1) was also used to calculate FPKMs (Fragments Per Kilo-base of exon per Million fragments mapped) of both lncRNAs and coding genes in each sample. FPKMs were calculated based on the length of the fragments and reads count mapped to this fragment. Gene FPKMs were computed by summing the FPKMs of transcripts in each gene group. Cuffdiff provides statistical routines for determining differential expression in digital transcript or gene expression data using a model based on the negative binomial distribution. The resulting *P*-values were adjusted with the Benjamini and Hochberg's approach for controlling the false discovery rate (FDR). Transcripts or genes with an adjusted *P*-value (*P*-adj) < 0.05 were assigned as differentially expressed (DE) between experimental groups (three biological replicates for each group).

Gene Ontology (GO) enrichment analysis of differentially expressed genes (DEGs) or lncRNA target genes was implemented by the GOseq R package, and GO terms with corrected *P* < 0.05 were considered significantly enriched by DEGs. KOBAS software was used to test the statistical enrichment of differentially expressed genes or lncRNA target genes in KEGG (Kyoto Encyclopedia of Genes and Genomes) pathways (http://www.genome.jp/kegg/).

### Quantitative Real-Time Polymerase Chain Reaction (qRT-PCR) Validation of Illumina Sequencing Data

To validate the Illumina sequencing data, 10 mRNA and ten lncRNA, which were selected from the most potential “lncRNA-mRNA” interactions based on the co-expression and co-localization analysis of DElncRNAs and DEmRNA among dietary groups, were tested for qRT-PCR analysis, using the same RNA samples for the transcriptome profiling. Specific primers were designed based on the data from GenBank (Table 3), and beta-2-microglobulin (β*-2-m*) was used as the reference gene according to our previous screening. The real-time PCR was carried out with SYBR Green Real-time PCR Master Mix (TaKaRa Biotechnology (Dalian) Co., Ltd., Dalian, China) in a quantitative thermal cycler (Mastercycler eprealplex, Eppendorf, German). The amplification efficiency for all primers, which was estimated by standard curves based on a 6-step 10-fold dilution series of target template, was within 95 ~105%, and the coefficients of linear regression (R^2^) were more than 0.99. The detailed program was similar with Xu et al. (2014). The mRNA expression levels were studied by qRT-PCR method: 2^−ΔΔCT^ (Livak and Schmittgen, 2001). The qRT-PCR data were subjected to one-way analysis of variance (ANOVA) in SPSS 16.0 (SPSS Inc., Chicago, USA) for Windows. Differences between means were tested by Tukey's multiple range test. The level of significance was chosen at *P* < 0.05, and the results were presented as means of triplicate tanks ± standard errors.

**Table 3:** Sequences of the primers used in this work.

**Primer**	**Sequence (5′-3′)**	**GenBank reference**	**Tm (°C)**	**Product length (bp)**
*baz2a*-F	GTCCCATTTCCAAACACGC	XM_017036014.1	58.3	120
*baz2a*-R	TGGACCTTAGGGCTTTTATGAG		58.4	
*kmt2d*-F	CCAGATGGAGGTGAAGACAGTC	XM_017035697.1	58.4	197
*kmt2d*-R	GCATAAAGCACAGGCGAAAT		58.2	
*mdtet3*-F	CAGCCCAATCTCAGGTATCCA	XM_017042774.1	59.9	183
*mdtet3*-R	CTTCGCACTTCGGGTCTAAAT		58.9	
*mthfd1*-F	TTCTTCCGTCTCATTTGGTCA	XM_008313708.1	58.0	158
*mthfd1*-R	CACCTGTAGAACCAGCAGACCT		58.5	
*rps10*-F	GATGCTGATGCCCAAGAAGA	XM_008320951.2	58.3	179
*rps10*-R	CCTTGACATACCCACAGGACTT		58.2	
*sfxn2*-F	TGAGAACGGGAACAAACTGG	XM_008314293.2	58.2	119
*sfxn2*-R	GCATGATGATGGGCAGAATAA		58.4	
*slcl6a5*-F	TACGCCACCGCTAACAACA	XM_008337600.2	58.3	182
*slcl6a5*-R	CAGATACTGTTACTGAGTCCGTTGA		58.4	
*usmg5*-F	AAAGGAGGGACTTCCGCTAA	XM_008317371.2	58.4	123
*usmg5*-R	AGAGTCGTGTCCACCCATGTT		58.9	
*zfp319*-F	AGCACCACTCGTCCCATAAC	XM_008311023.2	57.2	158
*zfp319*-R	TGGAAAAGGCAGAATAGAAGAAC		57.8	
*znf574*-F	TGTTTCTTCCTCTGGAGTCGC	XM_008322835.2	59.4	181
*znf574*-R	CATGCTCTGGATGAACCCTTT		59.1	
LNC_000230-F	AAACAACCTCCAGTAACCTTCC	novel	57.1	106
LNC_000230-R	TCTCTCTTGCGGGAGGACTA		57.3	
LNC_000255-F	GTTAGAGCACGACACGACCAA	novel	58.6	125
LNC_000255-R	GTAAAGCCAACCAACCGACA		58.4	
LNC_000285-F	TCAACCATCAGCGTACAGTAGG	novel	58.0	153
LNC_000285-R	GACGACGACGCTTTCACAAC		58.4	
LNC_000314-F	ACCTCCGACTGTTTGTATCACC	novel	58.7	155
LNC_000314-R	GTTCATCCTCTGCACTGGCT		57.3	
LNC_000360-F	GGAAATGAGTTCTGAAGTGCCT	novel	57.8	190
LNC_000360-R	TTCACCTGGCAGCAGTTTG		58.5	
LNC_000562-F	GCTGCCATTCCAAGACATACA	novel	58.5	114
LNC_000562-R	AAAAAGCCTGAGACACCCCT		57.9	
XR_521587.1-F	AGACTCTTGAATTGTCTGTTCATCC	XR_521587.2	58.7	158
XR_521587.1-R	CAGGGTGTTTGTTTATTTGTGC		57.7	
XR_521789.1-F	ACTTCGCCTCAGCCAATCA	XR_521789.2	58.7	95
XR_521789.1-R	AACCGTGTTCTCCATCAGCA		58.5	
XR_522182.1-F	TGGTAGCCGTTGACTTCCTT	XR_522182.2	57.3	104
XR_522182.1-R	TACTTTGACCTCTGCCTCATCTT		57.8	
XR_523541.1-F	CATGATGAGTGCTGGTGGCT	XR_523541.1	58.8	203
XR_523541.1-R	TGTGCTGCTGGTTGACATAGAG		58.8	
*β-2-m*-F	TTGGCTCGTGTTCGTCGTTC	XM_017034328.2	57.2	119
*β-2-m*-R	TCAGGGTGTTGGGCTTGTTG		58.3	

*Baz2a, bromodomain adjacent to zinc finger domain 2A; kmt2d, lysine (K)-specific methyltransferase 2D; mdtet3, methylcytosine dioxygenase TET3-like; mthfd1, methylenetetrahydrofolate dehydrogenase (NADP+ dependent) 1; rps10, ribosomal protein, S10 sfxn2, sideroflexin 2; slc16a5, solute carrier family 16 member 5; usmg5, up-regulated during skeletal muscle growth 5 homolog; zfp319, zinc finger protein 319-like; znf574, zinc finger protein 574; β-2-m, beta-2-microglobulin*.

### Availability of Materials

Detailed information about methods and materials used in the current study are available from the corresponding author on reasonable request.

## Results and Discussion

### Transcriptome Sequencing and Assembly

In the transcriptomic analysis, three pooled liver RNA samples were prepared for each dietary group (D/E-0.61, D/E-1.46, and D/E-2.75). Nine cDNA libraries were then constructed to perform Illumina sequencing. A total of 278,269,246, 269,325,362, and 279,307,386 clean reads were obtained for groups D/E-0.61, D/E-1.46, and D/E-2.75 respectively, giving rise to total clean bases of 41.74, 40.40 and 41.90 G, respectively (Supplementary Table 1). The average Q20 and Q30 (the sequencing error rate at 1 and 0.1% respectively) of the experimental samples was 96.17 and 90.17% respectively, indicating the high accuracy of the sequencing processes. Raw reads were deposited at the National Center for Biotechnology Information (NCBI)'s Sequence Read Archive under Accession No. SRP127310 (D1E2, D3E2, and D3E1 in the archived data represents groups D/E-0.61, D/E-1.46, and D/E-2.75 respectively). The reads were mapped on the genome of *C. semilaevis* and the average mapping rates of groups D/E-0.61, D/E-1.46, and D/E-2.75 was 77.62, 76.09, and 72.43%, respectively (Supplementary Table 2). The classification analysis of the reads showed that the total reals were comprised of (average): exon, 0.07%; mRNA, 67.72%; misc_RNA, 0.03%; ncRNA, 0.30%; tRNA, 1.19%; and others, 30.69% (Supplementary Table 3). Regarding the comparison among dietary groups, compared to groups D/E-1.46 and D/E-2.75, group D/E-0.61 had higher proportion of mRNA (70.21% vs. 66.52% and 66.43%), misc_RNA (0.04% vs. 0.02% and 0.02%), and ncRNA (0.41% vs. 0.27% and 0.22%). This indicated that dietary DHA/EPA ratio might have significant effects on transcription of different types of RNA.

### Filtration and Characterization of lncRNA

Large-scale analysis of the mammalian transcriptome have shown that the number and types of lncRNAs far exceed those of protein-coding mRNAs, while a small proportion of lncRNAs have been reported to have biological functions (Carninci et al., 2005; Birney et al., 2007; Carninci and Hayashizaki, 2007; Kapranov et al., 2007). In fish, however, very little information has been available about the identification and function prediction of lncRNAs. Only limited studies have been published in model fish such as zebrafish *Danio rerio* and rainbow trout *Oncorhynchus mykiss* (Kaushik et al., 2013; Liu et al., 2013; Haque et al., 2014; Dhiman et al., 2015; Al-Tobasei et al., 2016; Wang et al., 2016). Moreover, previous studies in both mammals and zebrafish have shown that lncRNAs are less conserved than protein-coding genes (Guttman et al., 2010; Cabili et al., 2011; Ulitsky et al., 2011; Derrien et al., 2012). Therefore, specific investigation is needed to identify and characterize the lncRNA profile in a certain fish species.

**Figure 1:**
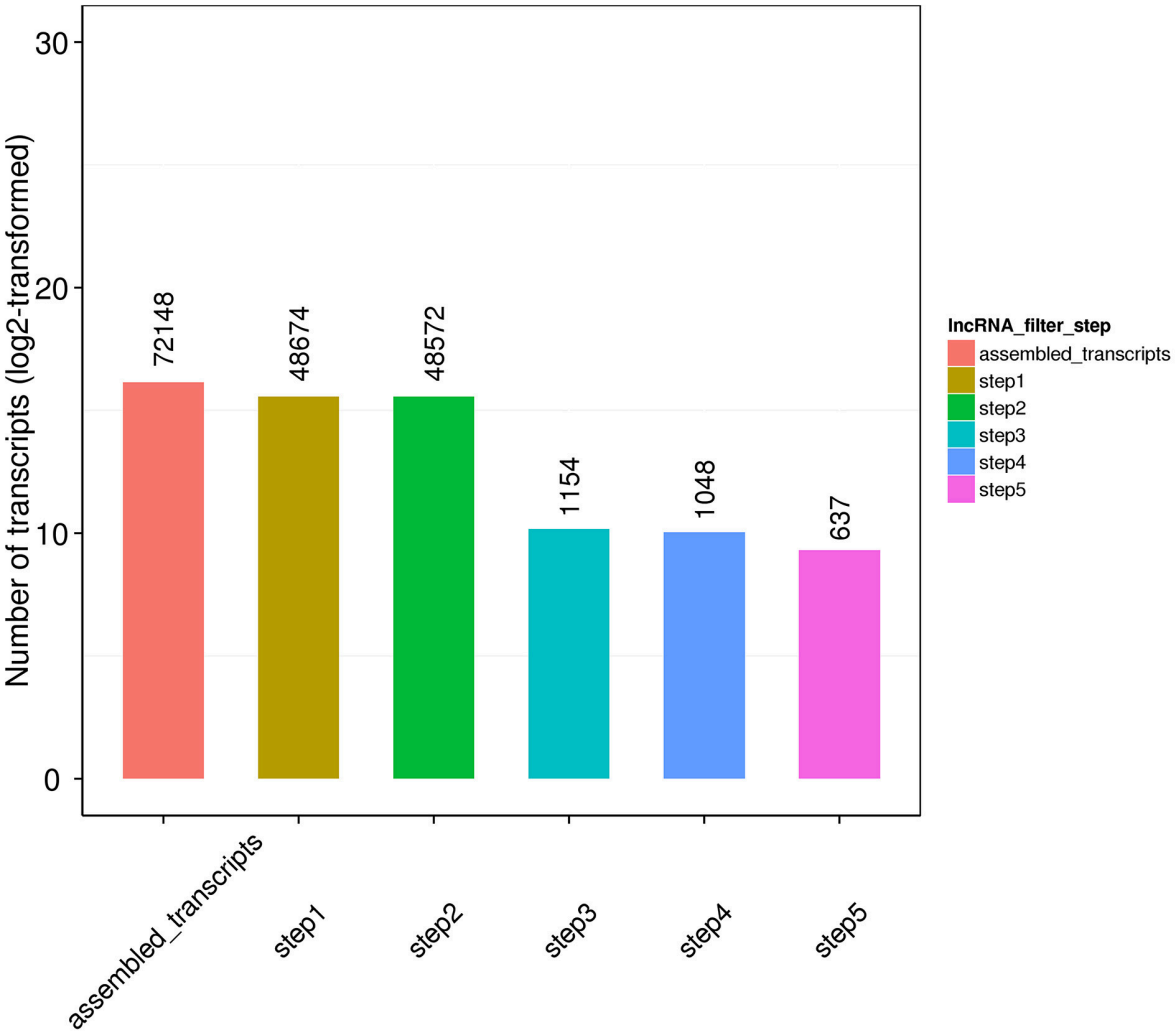
Filtration of lncRNA.

In the present study with tongue sole, based on the transcript assembly results, lncRNA was filtered following five steps: (1) number of exon (≥2); (2) length of transcript (>200bp); (3) comparison with annotated transcript (Cuffcompare software): transcripts overlapped with annotated coding exon were eliminated, and transcripts overlapped with annotated lncRNAs were regarded as annotated lncRNA; (4) expression level (Cuffquant software, FPKM≥0.5); (5) prediction of coding potential: transcripts containing no coding potential predicted by any mainstream software (CPC, CNCI, and PFAM) was recognized as novel lncRNAs (see Supplementary Figure 1 for the results), and transcripts with coding potential predicted by at least one software were designated as Transcripts of Uncertain Coding Potential (TUCP). The filtration results following these steps were presented in Figure 1.

**Figure 2:**
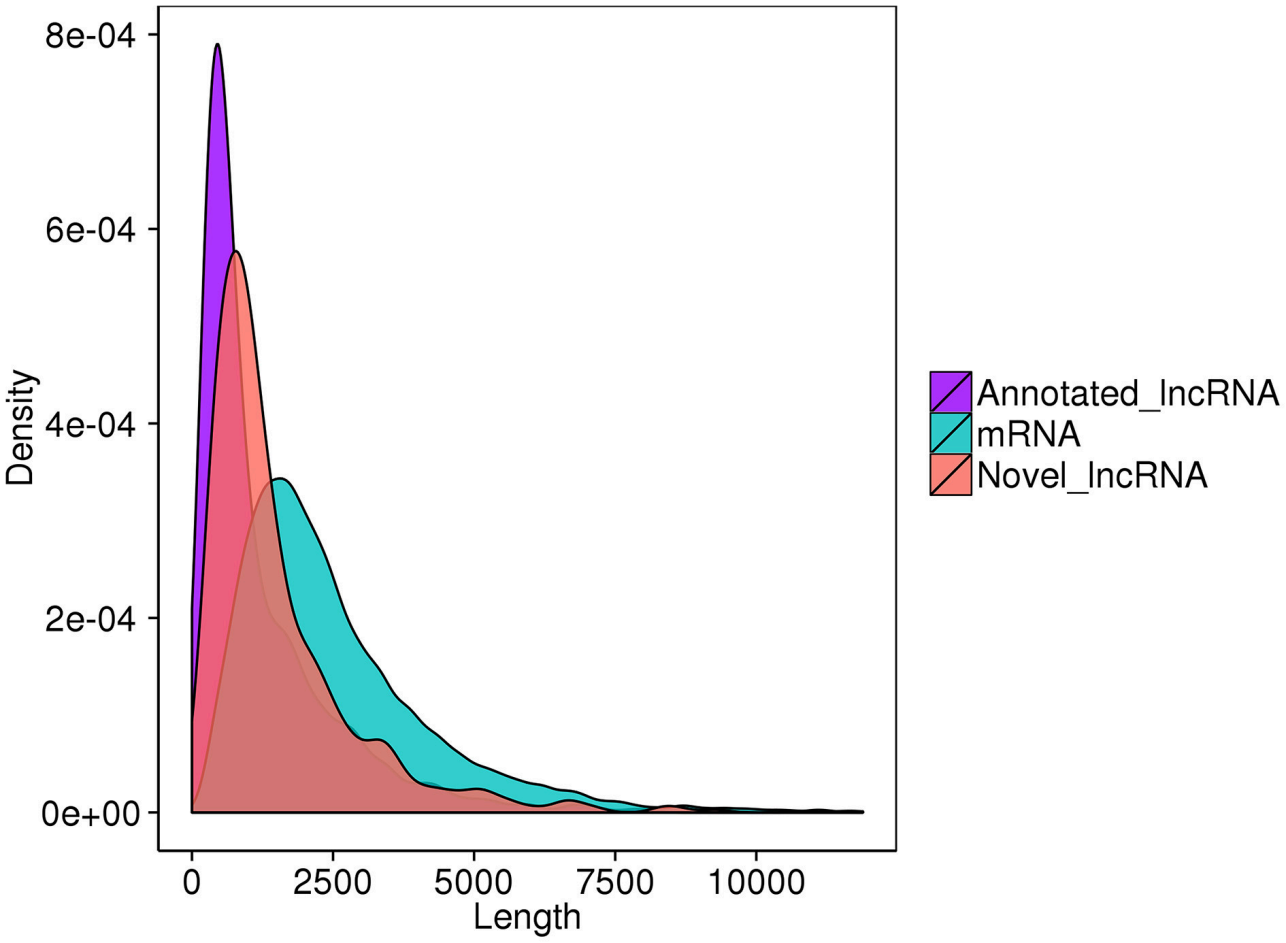
Length distribution of transcripts.

**Figure 3:**
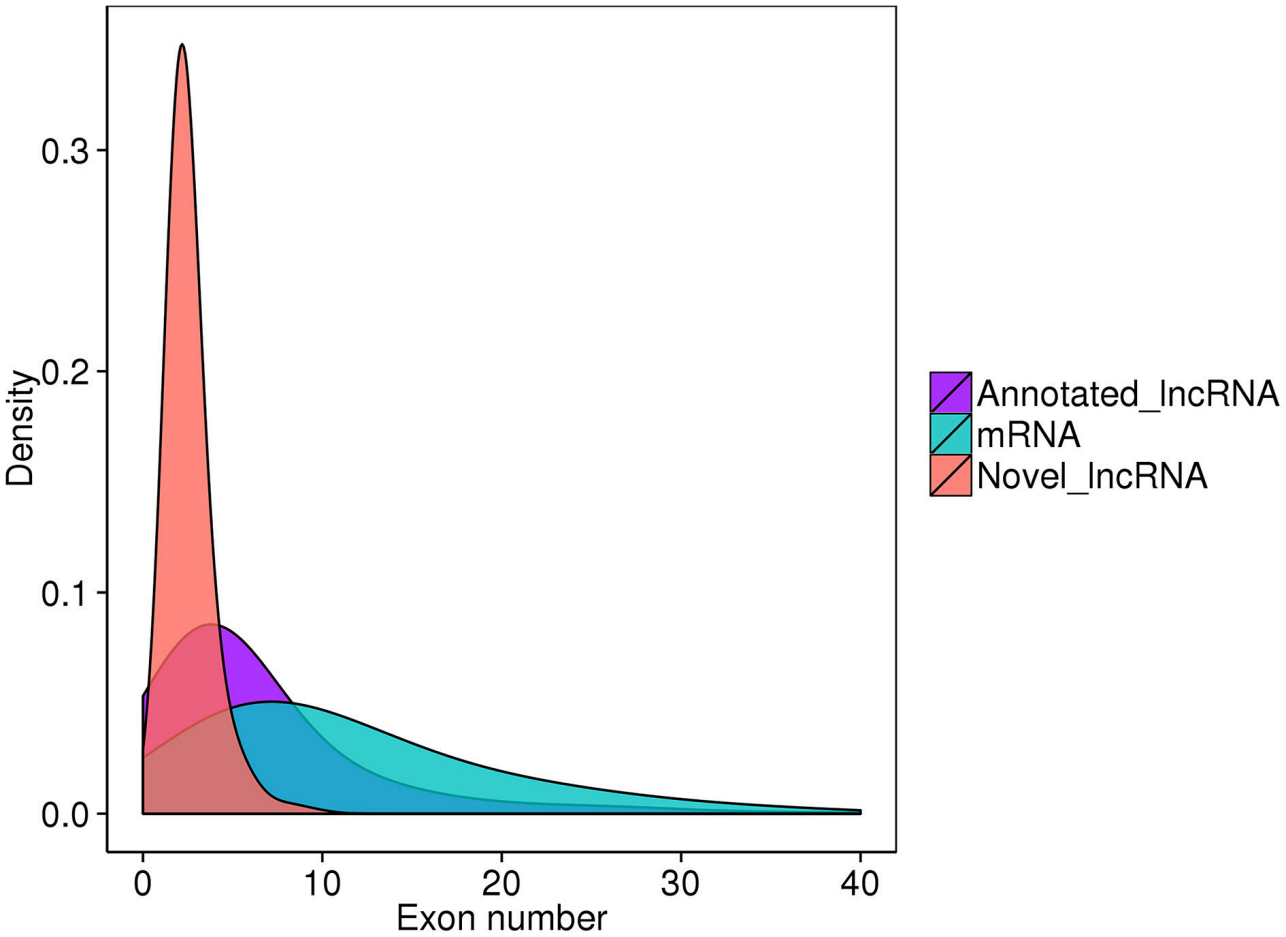
Exon distribution of transcripts.

**Figure 4:**
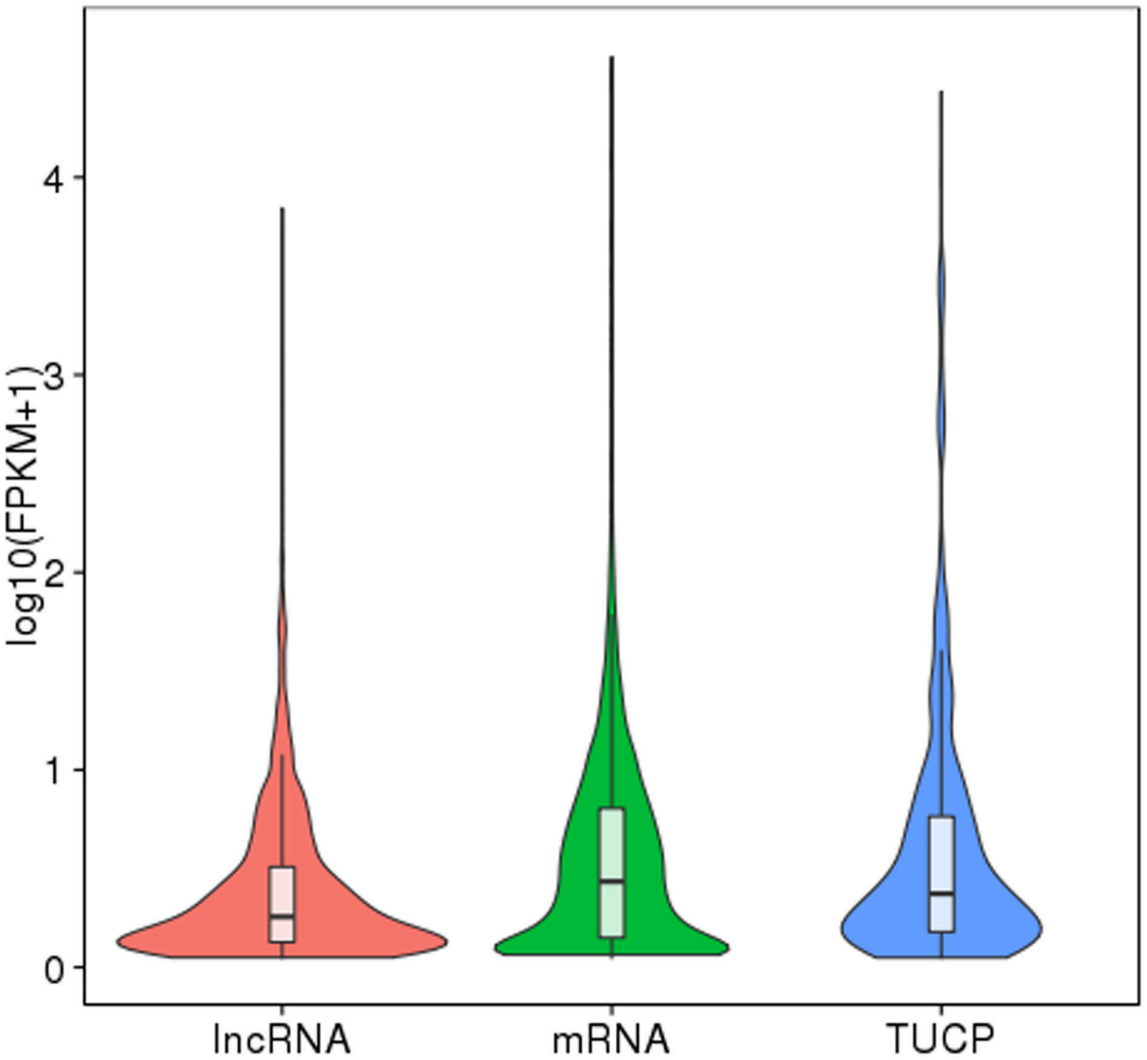
FPKM distribution of transcripts. The boxplot inside shows the FPKM distribution. The five characteristic values indicate maximum, upper quartile, mid-value, lower quartile, and minimum, respectively. The violin diagram shows the FPKM density distribution. The width of violin diagram represents the density of transcript under a certain expression level.

Finally, 789 annotated lncRNAs and 638 novel lncRNAs were obtained. These lncRNAs contained 69.9% lincRNA (long intergenic non-coding RNA), 30.1% antisense_lncRNA, but no intronic_lncRNA. Either annotated or novel lncRNAs were shorter and had less exons than mRNA (Figures 2, 3). Also, the average expression level of lncRNA was lower compared to mRNA (Figure 4). These characteristics of tongue sole lncRNAs were similar to those found in other species (Guttman et al., 2010; Cabili et al., 2011; Young and Ponting, 2013; Liang et al., 2015; Núñez-Acuña et al., 2017).

### Differentially Expressed Genes (DEGs) Among Dietary Groups

Non-coding RNAs have emerged as important regulators of cellular and systemic lipid metabolism (Chen, 2016; Zhou et al., 2016). In particular, the enigmatic class of long non-coding RNAs have been shown to play multifaceted roles in controlling transcriptional and posttranscriptional gene regulation (Kornfeld and Brüning, 2014; Gardini and Shiekhattar, 2015; Lopez-Pajares, 2016; Delás and Hannon, 2017; Long et al., 2017; Mathy and Chen, 2017).

**Figure 5:**
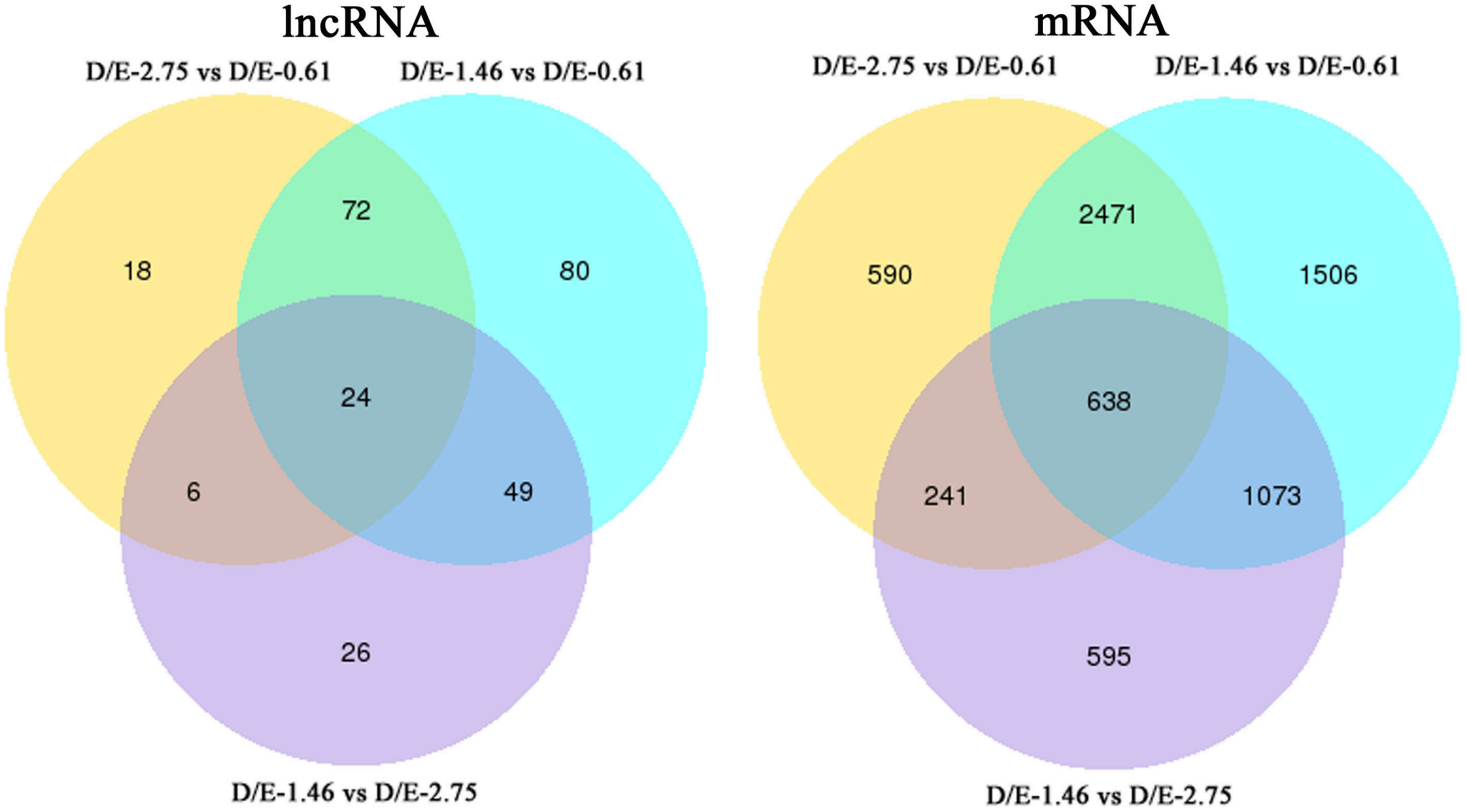
Venn diagram of differentially expressed genes.

In the present study, the experimental diets significantly affected the expression levels of both lncRNAs and mRNAs (Table 4). The most significant difference existed between groups D/E-0.61 and D/E-1.46. Compared to group D/E-0.61, D/E-1.46 significantly (adjusted *P* < 0.05) up-regulated expression of 178 lncRNA and 2629 mRNA, and down-regulated that of 47 lncRNA and 3059 mRNA. However, compared to group D/E-0.61, D/E-2.75 only significantly up-regulated expression of 78 lncRNA and 1925 mRNA, and down-regulated that of 42 lncRNA and 2015 mRNA. Generally, this result indicated that dietary DHA/EPA ratio regulated gene transcription in tongue sole in a dose-dependent manner. This was in accordance with the effects of dietary DHA/EPA ratio on growth performances and other physiological status such as lipid accumulation (Xu et al., 2018). As reported previously, compared to group D/E-0.61, D/E-1.46 but not D/E-2.75 significantly increased the growth rate and whole-body lipid content of juvenile tongue sole. In addition, despite the large number of differentially expressed (DE) genes among dietary groups, only 24 DElncRNA (about 16%) and 638 (about 16%) DEmRNA were overlapped among the three inter-group comparisons, D/E-1.46 vs. D/E-0.61, D/E-2.75 vs. D/E-0.61, and D/E-1.46 vs. D/E-2.75 (Figure 5). This indicated that each DHA/EPA ratio exerted a different regulation of the liver transcriptome. Moreover, the amount of DElncRNA was in proportion to that of DEmRNA among the inter-group comparisons. This suggested that the transcriptional activity of coding and non-coding RNAs in tongue sole liver was co-expressed in response to dietary DHA/EPA ratio, which was to be evidenced by the co-expression analysis described below.

**Table 4:** Differentially expressed genes among dietary groups.

**Compare**	**lncRNA**		**mRNA**		**TUCP**	
	**Up-**	**Down-**	**Up-**	**Down-**	**Up-**	**Down-**
D/E-1.46 vs. D/E-0.61	178	47	2629	3059	60	35
D/E-2.75 vs. D/E-0.61	78	42	1925	2015	37	35
D/E-1.46 vs. D/E-2.75	87	18	1073	1474	44	16

*TUCP, transcripts of uncertain coding potential*.

**Figure 6:**
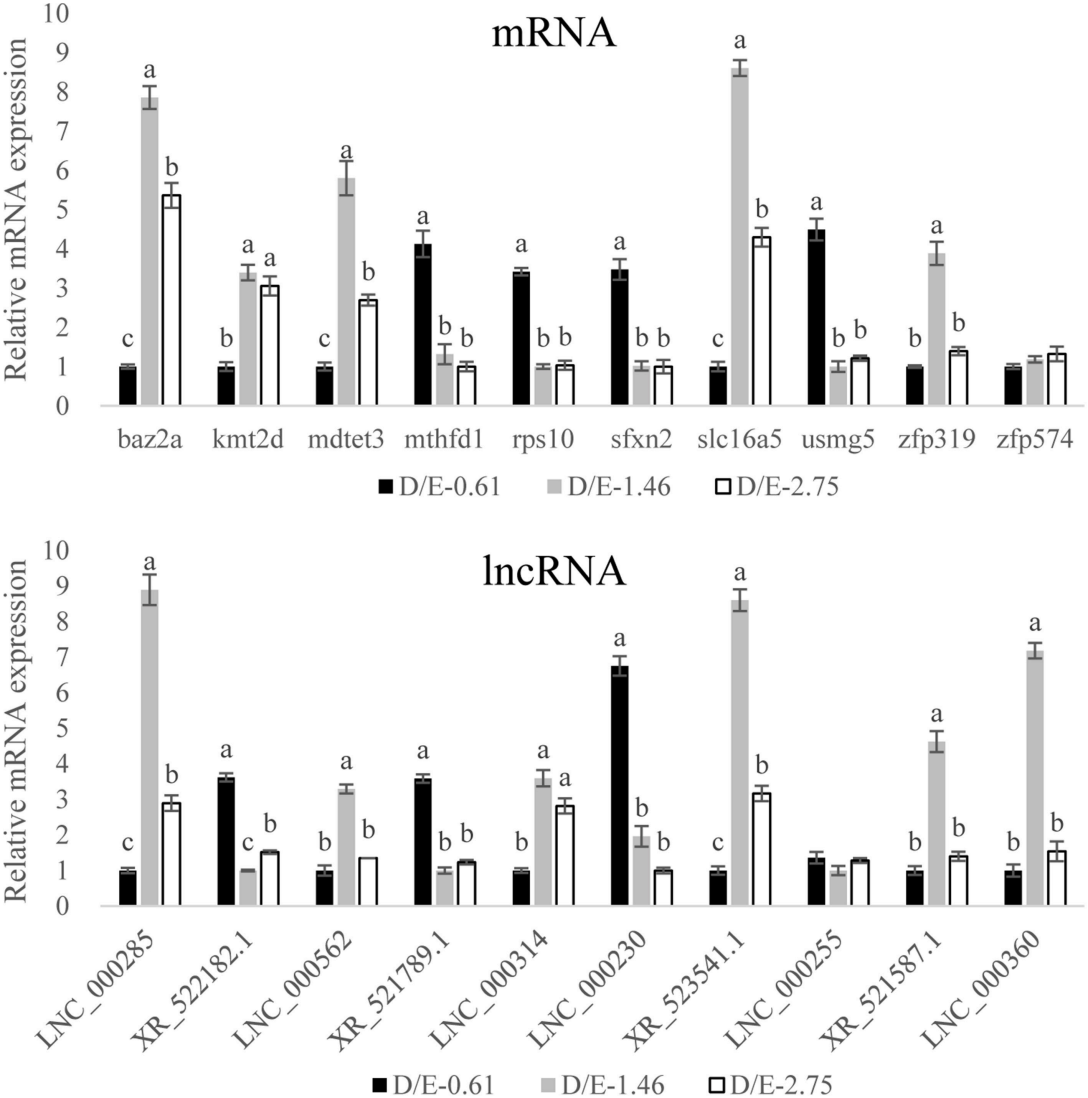
Validation of the transcriptome results by qRT-PCR measurement. The gene levels were expressed relative to β-2-microglobulin. Results are expressed as means ± standard error. For a certain mRNA or lncRNA, bars not sharing same letters denote significant (*P* < 0.05) difference in gene expression.

To confirm the DEG results from the transcriptomic assay, 10 mRNA and 10 lncRNA, which were selected from most potential “lncRNA-mRNA” interactions based on the co-expression and co-localization analysis of DElncRNA and DEmRNA described below, were tested for quantitative RT-PCR analysis. The results showed that transcription of 18 out of 20 selected genes was in good accordance with the transcriptomic results (Table 5, Figure 6). The validation results confirmed the high accuracy of the transcriptomic results.

**Table 5:** Transcriptome data of genes selected for qRT-PCR validation.

**Gene**	**Featured ID**	**Log**_**2**_**FC**			**Adjusted** ***P***		
		**D/E-1.46 vs. D/E-0.61**	**D/E-2.75 vs. D/E-0.61**	**D/E-1.46 vs. D/E-2.75**	**D/E-1.46 vs. D/E-0.61**	**D/E-2.75 vs. D/E-0.61**	**D/E-1.46 vs. D/E-2.75**
***mRNA***							
Bromodomain adjacent to zinc finger domain 2A (*baz2a*)	103385419	3.23	2.52	0.71	0.001	0.001	0.011
Lysine (K)-specific methyltransferase 2D (*kmt2d*)	103385102	1.86	1.73		0.001	0.001	
Methylcytosine dioxygenase TET3-like (*mdtet3*)	103397059	2.45	1.14	1.30	0.001	0.005	0.001
Methylenetetrahydrofolate dehydrogenase (NADP+ dependent) 1 (*mthfd1*)	103381395	−1.32	−1.25		0.001	0.001	
Ribosomal protein S10 (*rps10*)	103386602	−1.43	−1.27		0.001	0.001	
Sideroflexin 2 (*sfxn2*)	103381775	−1.31	−1.41		0.001	0.001	
Solute carrier family 16 member 5 (*slc16a5*)	103398853	2.04	0.92	1.13	0.001	0.005	0.001
Up-regulated during skeletal muscle growth 5 homolog (*usmg5*)	103384010	−2.17	−1.60		0.001	0.001	
Zinc finger protein 319-like (*zfp319*)	103379464	1.58		0.88	0.001		0.009
zinc finger protein 574 (*znf574*)	103387998	1.07			0.003		
***lncRNA***							
LNC_000230	XLOC_014945	−2.57	−3.41		0.001	0.001	
LNC_000255	XLOC_017426	−1.09	−0.80		0.003	0.041	
LNC_000285	XLOC_019682	3.37	2.26	1.11	0.010	0.049	0.037
LNC_000314	XLOC_021506	2.93	2.68		0.036	0.047	
LNC_000360	XLOC_024544	1.98	1.54		0.001	0.009	
LNC_000562	XLOC_038357	1.86		1.12	0.001		0.001
XR_521587.1	103379468	2.15		1.22	0.001		0.001
XR_521789.1	103381358	−1.11	−1.30		0.001	0.001	
XR_522182.1	103385094	−2.57	−1.66	–0.90	0.001	0.001	0.002
XR_523541.1	103398852	2.65	1.20	1.45	0.001	0.001	0.001

*FC, fold change*.

### Co-expression and Co-localization of lncRNA and mRNA

**Figure 7:**
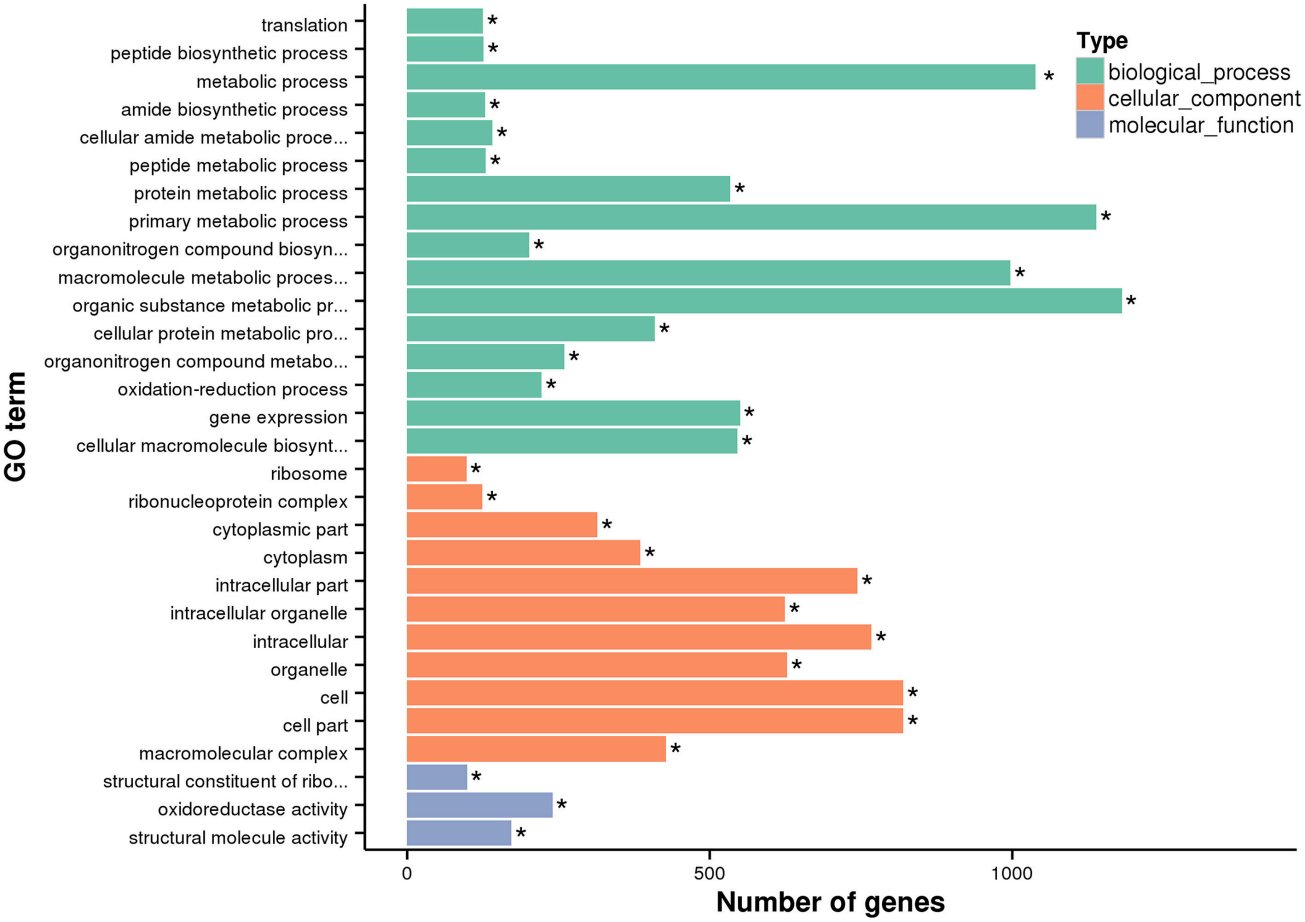
GO enrichment analysis for differentially expressed mRNA in lncRNA-mRNA co-expression analysis for D/E-1.46 vs. D/E-0.61. * denotes significant enrichment (*P*adj < 0.05, *P*adj is the adjusted *P*-value). The GO terms without full title: (1) Cellular amide metabolic process; (2) Organonitrogen compound biosynthetic process; (3) Macromolecule metabolic process; (4) Organic substance metabolic process; (5) Cellular protein metabolic process; (6) Organonitrogen compound metabolic process; (7) Cellular macromolecule biosynthetic process; (8) Structural constituent of ribosome.

**Figure 8:**
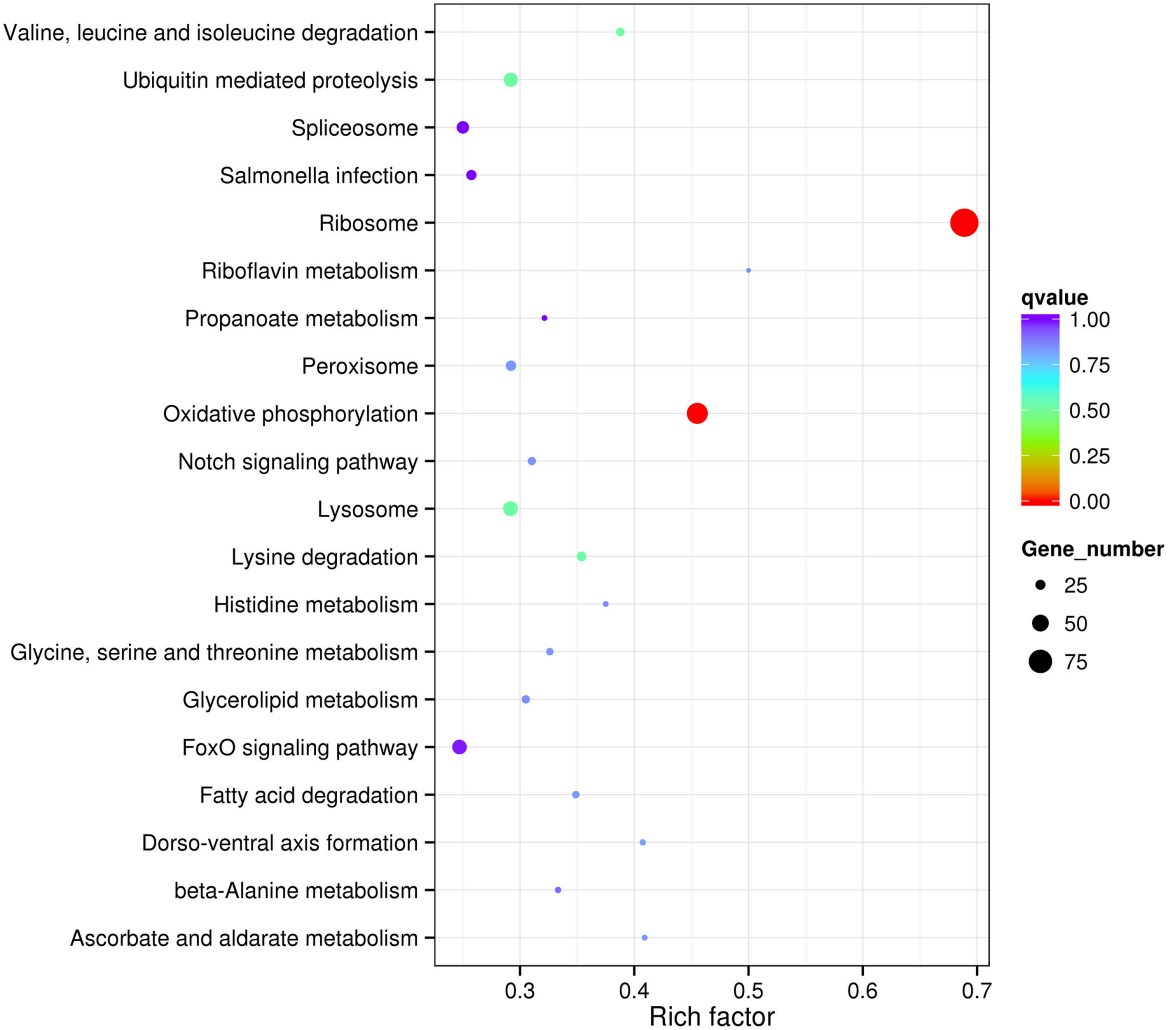
Statistics of KEGG pathway enrichment for differentially expressed mRNA in lncRNA-mRNA co-expression analysis for D/E-1.46 vs. D/E-0.61. Rich factor is the ratio of number of differentially expressed genes in a certain pathway to number of all annotated genes in this pathway. qvalue is corrected *P*-value by multiple hypothesis test. *q-*value < 0.05 denotes significant differences.

In order to estimate the potential lncRNA–mRNA interactions in response to dietary DHA/EPA ratio and consequently to predict the potential roles of the obtained lncRNAs in mRNA expression, co-expression and co-localization relationship of DElncRNA and DEmRNA among dietary groups were analyzed (Table 6). The co-expression analysis showed that 12888, 7659, and 2765 co-expression relationships were observed in inter-group comparisons D/E-1.46 vs. D/E-0.61, D/E-2.75 vs. D/E-0.61, and D/E-1.46 vs. D/E-2.75, respectively. The significant expression correlations between certain lncRNAs and protein-coding genes have also been observed in a previous study on rainbow trout investigating the intestinal lncRNA and coding RNAs transcription in response to functional diets (Núñez-Acuña et al., 2017). These results highlighted that the regulation of numerous protein-coding genes by dietary nutrients was highly correlated with lncRNAs. The GO enrichment analysis of the co-expression relationships showed that the most enriched GO terms were Metabolic process (including Protein metabolic process, Primary metabolic process, Macromolecule metabolic process, and Organic substance metabolic process), Gene expression, Cellular macromolecule bioynthetic process, Introcellular organelle (including Intracellular non-membrane-bounded organelle), and Oxidoreductase activity (see Figure 7 for D/E-1.46 vs. D/E-0.61, see Supplementary Figures 2A, S3A for D/E-2.75 vs. D/E-0.61, and D/E-1.46 vs. D/E-2.75, respectively). The KEGG pathway enrichment analysis of the co-expression relationships showed that the most enriched pathways were Ribosome and Oxidative phosphorylation (see Figure 8 for D/E-1.46 vs. D/E-0.61, see Supplementary Figures 2B,S3B for D/E-2.75 vs. D/E-0.61, and D/E-1.46 vs. D/E-2.75, respectively). These enrichment results were highly similar with the enrichment results for DEmRNA among experimental groups (Supplementary Figures 4,5), indicating the possible wide involvement of lncRNA in the regulation of mRNA expression. On the other hand, these results also indicated the effects of dietary DHA/EPA on a wide range of physiological processes in fish liver, which, however, was not the focus of the present study.

**Table 6:** Analysis of co-expression and co-localizaiton of differentially expressed lncRNA and mRNA among dietary groups.

**Comparison**	**Co-expression**	**Co-localization**	**Overlap**
D/E-1.46 vs. D/E-0.61	12,888	684	17
D/E-2.75 vs. D/E-0.61	7,659	278	13
D/E-1.46 vs. D/E-2.75	2,765	159	4
Overlap	761	14	2

As some lncRNAs could regulate the expression of coding genes near genomic regions, the co-localization of DElncRNA and DEmRNA among dietary groups were also analyzed. In total, 684, 278, and 159 co-localization relationships were observed in comparisons D/E-1.46 vs. D/E-0.61, D/E-2.75 vs. D/E-0.61, and D/E-1.46 vs. D/E-2.75, respectively. The co-localization relationships were primarily enriched in GO terms such as RNA metabolic process, Membrane-bounded organelle, Nucleus, RNA polymerase, and Nucleoside-triphosphatase regulator activity, as well as in KEGG pathways such as Ribosome and Glycerolipid metabolism (Supplementary Figures 6–8). Compared to the co-expression relationships, the co-localization relationships were more related to gene expression regulation, indicating the distance from target mRNA probably influenced the transcription-regulating effects of lncRNAs.

Considering that concurrent existence of co-expression and co-localization relationships of a certain lncRNA-mRNA match must increase the possibility of lncRNA-mRNA interaction, the overlap of co-expression and co-localization relationships of DElncRNA and DEmRNA was analyzed (Table 7). A total of 17, 13, and 4 overlapped lncRNA-mRNA matches was obtained for D/E-1.46 vs. D/E-0.61, D/E-2.75 vs. D/E-0.61, and D/E-1.46 vs. D/E-2.75, respectively. Two overlapped matches, “XR_523541.1–solute carrier family 16, member 5 (*slc16a5*)” and “LNC_000285–bromodomain adjacent to zinc finger domain 2A (*baz2a*)” were observed in all the three inter-group comparisons, indicating that they might play crucial roles in the effects of dietary DHA and EPA on hepatic gene transcription in tongue sole.

**Table 7:** Overlap of co-expression and co-localization relationships between differentially expressed lncRNAs and mRNAs among dietary groups.

**lncRNA_ID**	**mRNA**		**Co-expression**		**Co-location (lncRNA to mRNA)**	
	**ID**	**Gene name**	**Pearson correlation**	***P-*value**	**Distance**	**Location**
***D/E-1.46*** **vs**. ***D/E-0.61***						
XR_523541.1	103398853	Solute carrier family 16, member 5 (*slc16a5*)	0.990	3.82E-07	200	Downstream
XR_521587.1	103379464	Zinc finger protein 319-like (*zfp319*)	0.972	1.24E-05	19594	Upstream
XR_523068.1	103393985	Apolipoprotein A-IV-like (*apoa4*)	0.953	7.22E-05	67904	Downstream
XR_521789.1	103381395	Methylenetetrahydrofolate dehydrogenase (NADP+ dependent) 1 (*mthfd1*)	0.986	1.06E-06	54	Downstream
LNC_000230	103381775	Sideroflexin 2 (*sfxn2*)	0.961	3.79E-05	52923	Downstream
LNC_000230	103381769	Suppressor of cytokine signaling 3-like (*scs3*)	0.955	6.09E-05	191	Upstream
LNC_000285	103385419	Bromodomain adjacent to zinc finger domain 2A (*baz2a*)	0.958	4.60E-05	837	Downstream
LNC_000587	103398248	Chymotrypsin-like elastase family member 2A (*ce2a*)	0.970	1.46E-05	4443	Downstream
XR_522182.1	103385102	Lysine (K)-specific methyltransferase 2D (*kmt2d*)	−0.987	9.01E-07	74060	Downstream
XR_522818.1	103391927	Uncharacterized LOC103391927	0.957	5.37E-05	17005	Downstream
LNC_000360	103388031	Zinc finger protein 585A-like (*zfp585a*)	0.968	1.88E-05	67207	Downstream
LNC_000360	103387998	Zinc finger protein 574 (*zfp574*)	0.957	5.02E-05	98036	Upstream
LNC_000562	103397059	Methylcytosine dioxygenase TET3-like (*mdtet3*)	0.957	5.33E-05	746	Downstream
LNC_000314	103386594	Polyhomeotic-like protein 2 (*php2*)	0.959	4.23E-05	33381	Downstream
LNC_000314	103386602	Ribosomal protein S10 (*rps10*)	−0.968	1.87E-05	69929	Upstream
LNC_000255	103384010	Up-regulated during skeletal muscle growth 5 homolog (*usmg5*)	0.973	9.75E-06	91044	Downstream
XR_523487.1	103398370	Sodium/potassium/calcium exchanger 1-like (*spc1*)	0.951	8.46E-05	82748	Upstream
***D/E-2.75*** **vs**. ***D/E-0.61***						
XR_523541.1	103398853	Solute carrier family 16, member 5 (*slc16a5*)	0.990	3.82E-07	200	Downstream
XR_523068.1	103393985	Apolipoprotein A-IV-like (*apoa4*)	0.953	7.22E-05	67904	Downstream
XR_521789.1	103381395	Methylenetetrahydrofolate dehydrogenase (NADP+ dependent) 1 (*mthfd1*)	0.990	1.06E-06	54	Downstream
LNC_000230	103381775	Sideroflexin 2 (*sfxn2*)	0.961	3.79E-05	52923	Downstream
LNC_000230	103381769	Suppressor of cytokine signaling 3-like (*scs3*)	0.955	6.09E-05	191	Upstream
LNC_000285	103385419	Bromodomain adjacent to zinc finger domain 2A (*baz2a*)	0.958	4.60E-05	837	Downstream
LNC_000587	103398248	Chymotrypsin-like elastase family member 2A (*ce2a*)	0.952	7.35E-05	4443	Downstream
XR_522182.1	103385102	Lysine (K)-specific methyltransferase 2D (*kmt2d*)	−0.987	9.01E-07	74060	Downstream
XR_522818.1	103391927	Uncharacterized LOC103391927	0.957	5.37E-05	17005	Downstream
LNC_000360	103388031	Zinc finger protein 585A-like (*zfp585a*)	0.968	1.88E-05	67207	Downstream
LNC_000314	103386594	Polyhomeotic-like protein 2 (*php2*)	0.959	4.23E-05	33381	Downstream
LNC_000314	103386602	Ribosomal protein S10 (*rps10*)	−0.968	1.87E-05	69929	Upstream
LNC_000255	103384010	Up-regulated during skeletal muscle growth 5 homolog (*usmg5*)	0.973	9.75E-06	91044	Downstream
***D/E-1.46*** **vs**. ***D/E-2.75***						
XR_523541.1	103398853	Solute carrier family 16, member 5 (*slc16a5*)	0.990	3.82E-07	200	Downstream
XR_521587.1	103379464	Zinc finger protein 319-like (*zfp319*)	0.972	1.24E-05	19594	Upstream
LNC_000285	103385419	Bromodomain adjacent to zinc finger domain 2A (*baz2a*)	0.958	4.60E-05	837	Downstream
LNC_000562	103397059	Methylcytosine dioxygenase TET3-like (*mdtet3*)	0.957	5.33E-05	746	Downstream

Minus values in Pearson correlation denote negative correlation, i.e., “mRNA up-regulated but lncRNA down-regulated” or “mRNA down-regulated but lncRNA up-regulated.”

Solute carrier family 16 (SLC16) is a proton-linked monocarboxylate transporter (MCT), which comprises 14 members (Halestrap and Meredith, 2004), and mainly catalyzes the rapid transport of many monocarboxylates across the plasma membrane. While function of some members of this family such as SLC16A1 (also known as MCT1), SLC16A7 (MCT2), SLC16A8 (MCT3), and SLC16A3 (MCT4) have been elucidated (Bröer et al., 1997, 1998; Lin et al., 1998; Grollman et al., 2000; Manning Fox et al., 2000), functions of SLC16A5 (MCT6) are still unknown. Considering the possible roles of SLC16A5 in transmembrane transport of lactate, pyruvate, and ketone bodies, the significant difference in hepatic transcription of *slc16a5* among groups with different DHA/EPA ratios indicated that DHA and EPA may have different efficiency and priority in energy supply in tongue sole. The co-expression and co-localization relationship of XR_523541.1 and *slc16a5* suggested that the lncRNA XR_523541.1 may be involved in the regulation of *slc16a5* transcription by dietary DHA/EPA. Precise functions of both XR_523541.1 and *slc16a5* need to be elucidated by future studies.

Baz2a is an essential component of nucleolar remodeling complex, a complex that mediates silencing of a fraction of rDNA by recruiting histone-modifying enzymes and DNA methyltransferases, leading to heterochromatin formation and transcriptional silencing (Gu et al., 2015). The co-expression and co-localization relationship of “LNC_000285–*baz2a*” indicated that the lncRNA LNC_000285 probably mediated the regulatory effects of dietary DHA/EPA on gene expression through promoting heterochromatin formation. Inducing heterochromatin formation and suppressing mobile elements were major mechanisms of transcription-regulating effects of non-coding RNAs (Cusanelli and Chartrand, 2015; Meller et al., 2015; Acharya et al., 2017; da Rocha and Heard, 2017; Oliva-Rico and Herrera, 2017). In human pancreatic ductal adenocarcinoma, the lncRNA HOTAIR contributed in the silence of MicroRNA-34a by enchancer of zeste homolog 2 through induction of heterochromatin formation (Li et al., 2016). Microsatellite repeat DXZ4-associated long non-coding RNAs also had developmental changes in expression coincident with heterochromatin formation at the human (*Homo sapiens*) macrosatellite repeat (Figueroa et al., 2015). Other evidence showed that lncRNA maturation to initiate heterochromatin formation in the nucleolus is required in exit from pluripotency in embryonic stem cells (Savić et al., 2014).

Besides “LNC_000285–*baz2a*,” the overlapped matches “LNC_000562–methylcytosine dioxygenase TET3-like (*mdtet3*)” and “LNC_000314–polyhomeotic-like protein 2 (*php2*)” also provided potential evidence for the roles of lncRNAs in chromatin remodeling. Mdtet3 catalyzes DNA demethylation and thus plays important roles in epigenetic chromatin reprogramming and maintenance of genome stability (Xu et al., 2012; Deplus et al., 2013; Jiang et al., 2017). Php2 is a component of a Polycomb group (PcG) multiprotein PRC1-like complex, which is required to maintain the transcriptionally repressive state of many genes, and acts via chromatin remodeling and modification of histones (Isono et al., 2005).

The overlapped match “XR_522182.1–lysine (K)-specific methyltransferase 2D (*kmt2d*)” was observed in inter-group comparisons D/E-2.75vs. D/E-0.61 and D/E-1.46vs. D/E-0.61. As a histone methyltransferase, Kmt2d methylates 'Lys-4' of histone H3 into H3K4me, which represents a specific tag for epigenetic transcriptional activation (Cho et al., 2007; Lan et al., 2007). This result indicated the potential roles of lncRNA in epigenetic transcriptional regulation.

Other mechanisms of transcription-regulating effects of lncRNAs were also indicated from the present results. For example, several overlapped “lncRNA–zinc finger protein” matches, such as “XR_521587.1–zinc finger protein 319-like (*zfp319*),” “LNC_000360–zinc finger protein 574 (*zfp574*),” and “LNC_000360–zinc finger protein 585A-like (*zfp585a*),” were observed in the co-expression and co-localization analysis of DElncRNAs and DEmRNAs. Interacting with these zinc finger proteins may be a possible way of lncRNA functioning on gene expression.

Interactions of other lncRNAs with some mRNAs observed in overlapped “lncRNA–mRNA” matches of the current analysis have been reported in human and terrestrial animal studies. For example, the lncRNA–mRNA match “XR_523068.1–apolipoprotein A-IV (*apoa4*)” was overlapped in the co-expression and co-localization analysis in the present study. A study on mice has shown that an anti-sense lncRNA (APOA4-AS) concordantly and specifically regulated *apoa4* expression both *in vitro* and *in vivo* with the involvement of the mRNA stabilizing protein HuR (Qin et al., 2016). The alignment analysis did not find homology between mice APOA4-AS and tongue sole XR_523068.1. Moreover, mice APOA4-AS was transcribed from the opposite strand of *apoa4* locus (+ strand), and partially overlapped with *apoa4* exon 3 and 3'-UTR, while tongue sole XR_523068.1 was far away from tongue sole *apoa4* (67904 bp downstream). In mice, gene expression of another apolipoprotein APOC2 was also reported to be regulated by the lncRNA lncLSTR (Li et al., 2015). These results indicated that lncRNAs may play certain roles in gene expression of apolipoproteins, which are key components of lipoproteins and play important roles in lipid transportation. In addition, the mRNA expression of *apoa4* in response to dietary DHA/EPA ratio was negatively correlated with the hepatic lipid content of the experimental fish (Xu et al., 2018). The lower hepatic *apoa4* expression in group D/E-1.46 may reduce the transportation of lipid out of liver, and thus contribute to higher hepatic lipid accumulation in this group.

Interactions between lncRNAs and suppressor of cytokine signaling 3-like (*scs3*) or methylenetetrahydrofolate dehydrogenase (NADP+ dependent) (*mthfd*) have also been observed in human studies. A recent study on postmenopausal osteoporosis observed a probable interaction between lncRNA LINC00963 and *scs3* based on analysis of DElncRNA–DEmRNA co-expression network (Fei et al., 2018). However, different from the present study which showed that lncRNA LNC_000230 was 191 bp upstream relative to co-expressed *scs3*, in that study LINC00963 and *scs3* were located in different chromosomes. Regarding *mthfd*, a study with MCF-7 breast cancer cells showed that a lncRNA, taurine-upregulated gene 1 (*tug1*), positively regulated the gene expression of *mthfd2* (Zhao and Ren, 2016). *Tug1* and *mthfd2* were also located in different chromosomes, while in the present study, lncRNA XR_521789.1 was 54 bp downstream relative to co-expressed *mthfd1*.

Other overlapped “lncRNA–mRNA” matches in the co-express and co-localization analysis, such as “LNC_000587 - chymotrypsin-like elastase family member 2A (*ce2a*),” “LNC_000230–sideroflexin 2 (*sfxn2*),” “XR_523487.1–sodium/potassium/calcium exchanger 1-like (*spc1*),” “LNC_000314–ribosomal protein S10 (*rps10*),”and “LNC_000255–up-regulated during skeletal muscle growth 5 homolog (*usmg5*),”were novel findings of the present study. No similar or relevant lncRNA-mRNA interactions have been reported in other studies. These findings provided useful sources for further investigation on lncRNA–mRNA interactions, especially regarding the effects of dietary fatty acids.

In conclusion, this was the first time in marine teleost to investigate the possible lncRNA-mRNA interactions in response to dietary fatty acids. The results provided novel knowledge of lncRNAs in non-model marine teleost, and will serve as important resources for future studies that further investigate the roles of lncRNAs in lipid metabolism of marine teleost. Potential lncRNA–mRNA interactions “XR_523541.1–*slc16a5*” and “LNC_000285–*baz2a*” may play important roles in effects of dietary DHA/EPA on hepatic gene transcription in tongue sole.

### Non-standard Abbreviations

DE, differentially expressed; DEG: differentially expressed gene; *slc16a5*, solute carrier family 16, member 5; *baz2a*: bromodomain adjacent to zinc finger domain 2a; *mct*, monocarboxylate transporter; *mdtet3*, methylcytosine dioxygenase tet3-like; *php2*, polyhomeotic-like protein 2; *kmt2d*, lysine (k)-specific methyltransferase 2d; *zfp*, zinc finger protein; *apo*, apolipoprotein; *scs3*, suppressor of cytokine signaling 3-like; *mthfd1*, methylenetetrahydrofolate dehydrogenase (nadp+ dependent) 1; *ce2a*, chymotrypsin-like elastase family member 2a, *sfxn2*; sideroflexin 2; *spc1*, sodium/potassium/calcium exchanger 1-like; *rps10*, ribosomal protein s10; *usmg5*, up-regulated during skeletal muscle growth 5 homolog.

## Data Availability

The raw reads obtained from the transcriptomic analysis have been deposited at the National Center for Biotechnology Information (NCBI)'s Sequence Read Archive under Accession No. SRP127310. Other raw data supporting the conclusions of this manuscript will be made available by the authors, without undue reservation, to any qualified researcher.

## Author Contributions

HX and ML designed the study and wrote the manuscript. LC conducted the feeding trial. HX, LC, and BS did the transcriptomic analysis. LC and YW did the qRT-PCR study. All authors read and approved the final version of the manuscript.

### Conflict of Interest Statement

The authors declare that the research was conducted in the absence of any commercial or financial relationships that could be construed as a potential conflict of interest.
