# Supplementary material for: Transcriptomic Analysis of Potential “lncRNA–mRNA” Interactions in Liver of the Marine Teleost Cynoglossus semilaevis Fed Diets With Different DHA/EPA Ratios
Source: Front Physiol. 2019 Apr 2;10:331. doi: 10.3389/fphys.2019.00331 (PMC6454198; doi:10.3389/fphys.2019.00331)
Supplement: Supplementary file 1 [file Table_1.docx]

**SUPPLEMENTARY INFORMATION**

**Transcriptomic analysis of potential “lncRNA-mRNA” interactions in liver of the marine teleost** ***Cynoglossus semilaevis* fed diets with different DHA/EPA ratios**

*Houguo Xu, Lin Cao, Bo Sun, Yuliang Wei, Mengqing Liang**

*Correspondence: Mengqing Liang, Yellow Sea Fisheries Research Institute, Chinese Academy of Fishery Sciences, 106 Nanjing Road, Qingdao 266071, China

e-mail: [liangmq@ysfri.ac.cn](mailto:liangmq@ysfri.ac.cn)

**Supplementary Table S1** Clean data and quality control. Q20 and Q30: the sequencing error rate 0.01 and 0.001 respectively.

| Sample | Raw Reads | Clean Reads | Clean Bases | Error (%) | Q20 (%) | Q30 (%) | GC Content (%) |
| --- | --- | --- | --- | --- | --- | --- | --- |
| D/E-0.61_1 | 107,114,354 | 91,101,546 | 13.67 G | 0.02 | 95.90 | 89.57 | 52.64 |
| D/E-0.61_2 | 97,670,436 | 87,678,192 | 13.15 G | 0.02 | 96.06 | 89.70 | 52.73 |
| D/E-0.61_3 | 109,633,872 | 99,489,508 | 14.92 G | 0.02 | 96.11 | 89.82 | 52.65 |
| D/E-1.46_1 | 102,084,264 | 95,811,190 | 14.37 G | 0.02 | 95.96 | 89.58 | 51.98 |
| D/E-1.46_2 | 88,929,330 | 83,187,382 | 12.48 G | 0.02 | 95.79 | 89.67 | 50.89 |
| D/E-1.46_3 | 97,211,738 | 90,326,790 | 13.55 G | 0.02 | 96.06 | 89.75 | 51.49 |
| D/E-2.75_1 | 100,870,722 | 91,701,036 | 13.76 G | 0.02 | 96.59 | 91.23 | 49.23 |
| D/E-2.75_2 | 106,350,278 | 97,266,618 | 14.59 G | 0.02 | 96.61 | 91.36 | 48.19 |
| D/E-2.75_3 | 98,281,128 | 90,339,732 | 13.55 G | 0.02 | 96.42 | 90.85 | 49.08 |

**Supplementary Table S2** Reads mapping to the reference genome.

| Sample | D/E-0.61_1 | D/E-0.61_2 | D/E-0.61_3 | D/E-1.46_1 | D/E-1.46_2 | D/E-1.46_3 | D/E-2.75_1 | D/E-2.75_2 | D/E-2.75_3 |
| --- | --- | --- | --- | --- | --- | --- | --- | --- | --- |
| Total reads | 91,101,546 | 87,678,192 | 99,489,508 | 95,811,190 | 83,187,382 | 90,326,790 | 91,701,036 | 97,266,618 | 90,339,732 |
| Total mapped | 64,248,144 (70.52%) | 70,766,754 (80.71%) | 81,205,244 (81.62%) | 74,479,491 (77.74%) | 58,678,498 (70.54%) | 72,251,790 (79.99%) | 64,308,327 (70.13%) | 69,031,436 (70.97%) | 68,835,315 (76.2%) |
| Multiple mapped | 2,723,284 (2.99%) | 3,277,007 (3.74%) | 3,868,162 (3.89%) | 2,958,993 (3.09%) | 2,349,699 (2.82%) | 2,652,563 (2.94%) | 2,804,579 (3.06%) | 2,827,340 (2.91%) | 3,079,781 (3.41%) |
| Uniquely mapped | 61,524,860 (67.53%) | 67,489,747 (76.97%) | 77,337,082 (77.73%) | 71,520,498 (74.65%) | 56,328,799 (67.71%) | 69,599,227 (77.05%) | 61,503,748 (67.07%) | 66,204,096 (68.06%) | 65,755,534 (72.79%) |
| Read-1 | 31,746,465 (34.85%) | 34,672,980 (39.55%) | 39,850,178 (40.05%) | 36,836,642 (38.45%) | 29,622,302 (35.61%) | 35,767,697 (39.6%) | 31,427,397 (34.27%) | 33,737,074 (34.69%) | 33,760,293 (37.37%) |
| Read-2 | 29,778,395 (32.69%) | 32,816,767 (37.43%) | 37,486,904 (37.68%) | 34,683,856 (36.2%) | 26,706,497 (32.1%) | 33,831,530 (37.45%) | 30,076,351 (32.8%) | 32,467,022 (33.38%) | 31,995,241 (35.42%) |
| Reads map to '+' | 30,565,481 (33.55%) | 33,493,673 (38.2%) | 38,385,670 (38.58%) | 35,487,423 (37.04%) | 27,930,554 (33.58%) | 34,565,132 (38.27%) | 30,633,396 (33.41%) | 32,913,242 (33.84%) | 32,706,271 (36.2%) |
| Reads map to '-' | 30,959,379 (33.98%) | 33,996,074 (38.77%) | 38,951,412 (39.15%) | 36,033,075 (37.61%) | 28,398,245 (34.14%) | 35,034,095 (38.79%) | 30,870,352 (33.66%) | 33,290,854 (34.23%) | 33,049,263 (36.58%) |
| Non-splice reads | 32,878,079 (36.09%) | 31,891,393 (36.37%) | 36,010,279 (36.2%) | 37,849,002 (39.5%) | 29,392,599 (35.33%) | 36,074,383 (39.94%) | 34,487,810 (37.61%) | 37,481,666 (38.53%) | 33,012,210 (36.54%) |
| Splice reads | 28,646,781 (31.44%) | 35,598,354 (40.6%) | 41,326,803 (41.54%) | 33,671,496 (35.14%) | 26,936,200 (32.38%) | 33,524,844 (37.12%) | 27,015,938 (29.46%) | 28,722,430 (29.53%) | 32,743,324 (36.24%) |
| Reads mapped in proper pairs | 55,254,108 (60.65%) | 61,360,272 (69.98%) | 70,585,464 (70.95%) | 64,908,512 (67.75%) | 47,798,318 (57.46%) | 63,484,840 (70.28%) | 55,467,932 (60.49%) | 60,176,334 (61.87%) | 58,973,896 (65.28%) |
| Proper-paired reads map to different chrom | 12 (0%) | 10 (0%) | 16 (0%) | 26 (0%) | 62 (0%) | 56 (0%) | 68 (0%) | 86 (0%) | 154 (0%) |

**Supplementary Table S3** Classification of mapped reads.

| Sample | D/E-0.61_1 | D/E-0.61_2 | D/E-0.61_3 | D/E-1.46_1 | D/E-1.46_2 | D/E-1.46_3 | D/E-2.75_1 | D/E-2.75_2 | D/E-2.75_3 |
| --- | --- | --- | --- | --- | --- | --- | --- | --- | --- |
| exon | 21,592 (0.08%) | 22,378 (0.08%) | 21,117 (0.08%) | 21,239 (0.07%) | 20,326 (0.08%) | 29,813 (0.10%) | 16,732 (0.06%) | 22,237 (0.08%) | 30,549 (0.09%) |
| mRNA | 17,456,817 (68.57%) | 19,009,603 (68.69%) | 20,223,102 (73.36%) | 19,246,858 (63.39%) | 16,537,866 (67.74%) | 20,458,404 (68.44%) | 17,075,945 (65.11%) | 19,686,950 (67.02%) | 22,466,218 (67.16%) |
| misc_RNA | 10,478 (0.04%) | 10,317 (0.04%) | 9,692 (0.04%) | 7,568 (0.02%) | 7,334 (0.03%) | 7,442 (0.02%) | 6,933 (0.03%) | 4,306 (0.01%) | 4,822 (0.01%) |
| ncRNA | 112,090 (0.44%) | 119,421 (0.43%) | 98,017 (0.36%) | 73,555 (0.24%) | 72,855 (0.30%) | 78,830 (0.26%) | 77,353 (0.29%) | 55,872 (0.19%) | 62,128 (0.19%) |
| rRNA | 0 (0.00%) | 0 (0.00%) | 0 (0.00%) | 0 (0.00%) | 0 (0.00%) | 0 (0.00%) | 0 (0.00%) | 0 (0.00%) | 0 (0.00%) |
| tRNA | 521,328 (2.05%) | 618,445 (2.23%) | 330,730 (1.20%) | 245,595 (0.81%) | 268,404 (1.10%) | 271,984 (0.91%) | 363,027 (1.38%) | 140,318 (0.48%) | 170,586 (0.51%) |
| Others | 7,335,370 (28.81%) | 7,894,218 (28.53%) | 6,884,769 (24.97%) | 10,766,495 (35.46%) | 7,507,037 (30.75%) | 9,048,035 (30.27%) | 8,687,029 (33.12%) | 9,463,204 (32.22%) | 10,717,833 (32.04%) |

**
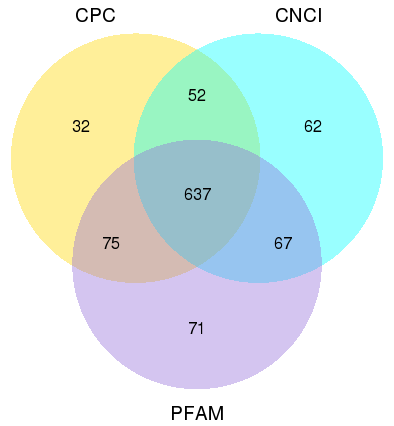
**

**Supplementary Fig. S1** **Number of transcripts with coding potential predicted by different softwares.**

(A)


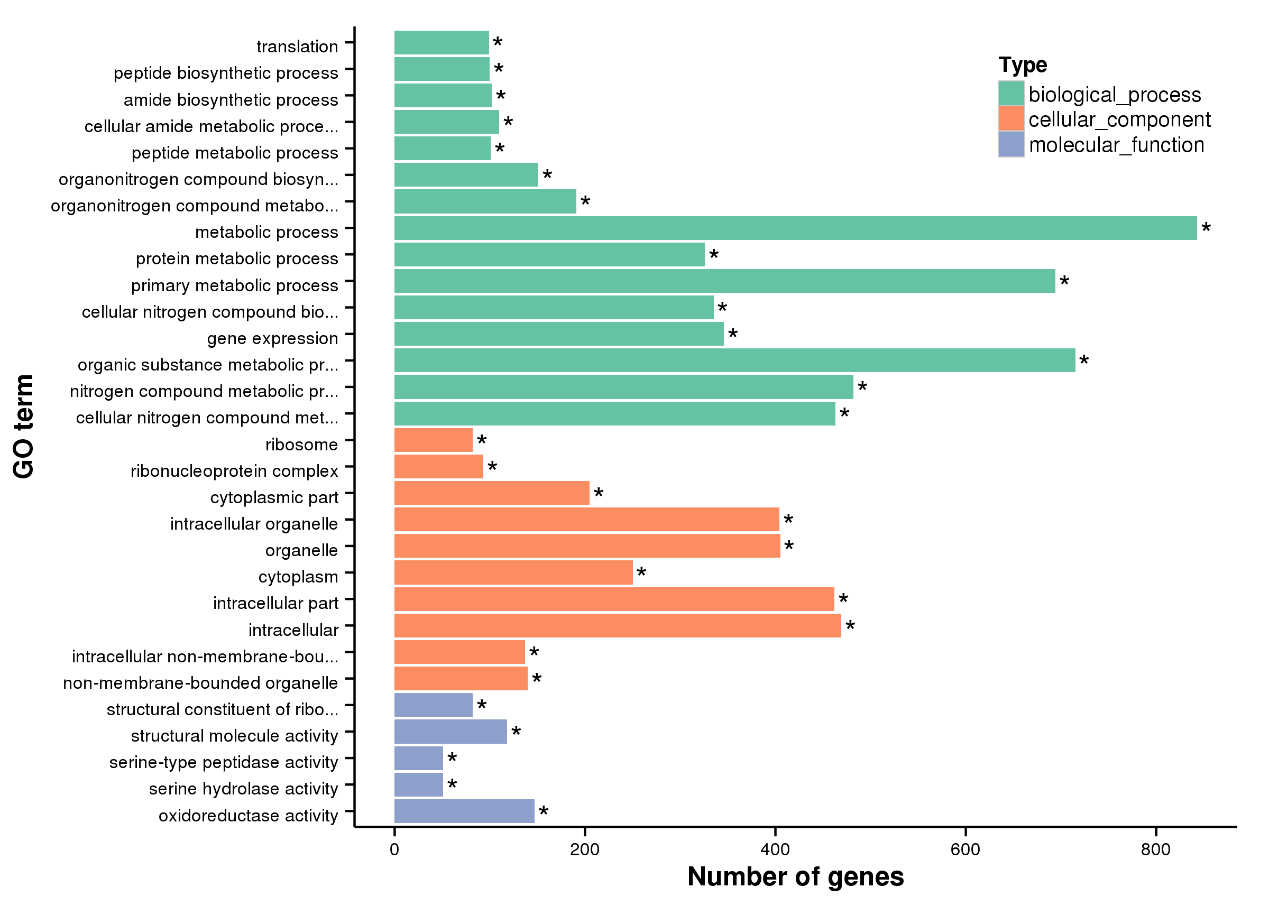


(B)


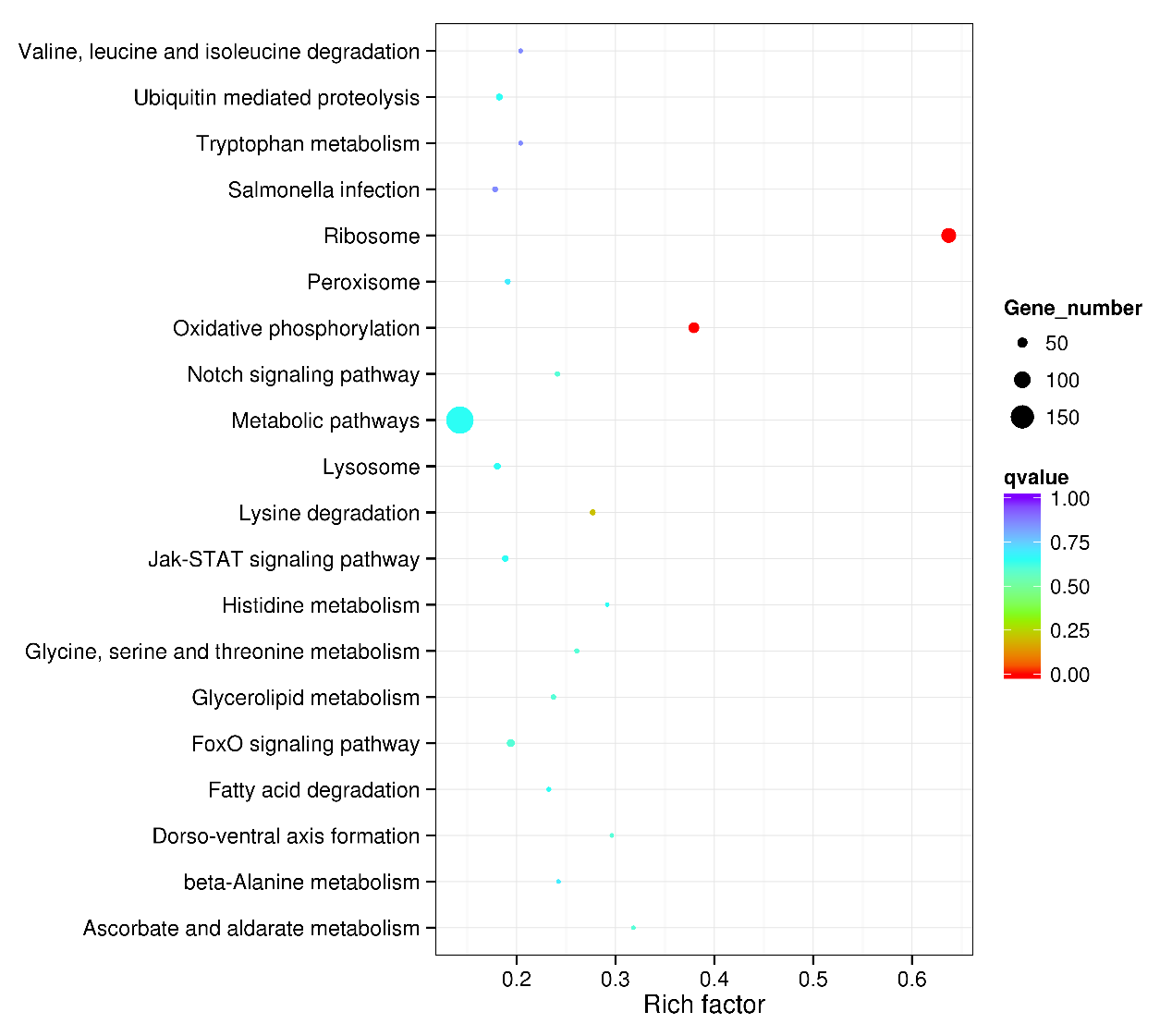


**Supplementary Fig. S2** **GO enrichment analysis (A) and statistics of KEGG pathway enrichment (B) for differentially expressed mRNA in lncRNA-mRNA co-expression analysis for D/E-2.75 vs D/E-0.61.** * denotes significant enrichment (*P*adj < 0.05, *P*adj is the adjusted *P*-value). The GO terms without full title: 1) Cellular amide metabolic process; 2) Organonitrogen compound biosynthetic process; 3) Cellular nitrogen compound biosynthetic process; 4) Organic substance metabolic process; 5) Nitrogen compound metabolic process; 5) Cellular nitrogen compound metabolic process; 6) Intracellular non-membrane-bounded organelle; 7) Non-membrane-bounded organelle; 8) Structural constituent of ribosome. Rich factor is the ratio of number of differentially expressed genes in a certain pathway to number of all annotated genes in this pathway. qvalue is corrected *P* value by multiple hypothesis test. qvalue < 0.05 denotes significant differences.

(A)


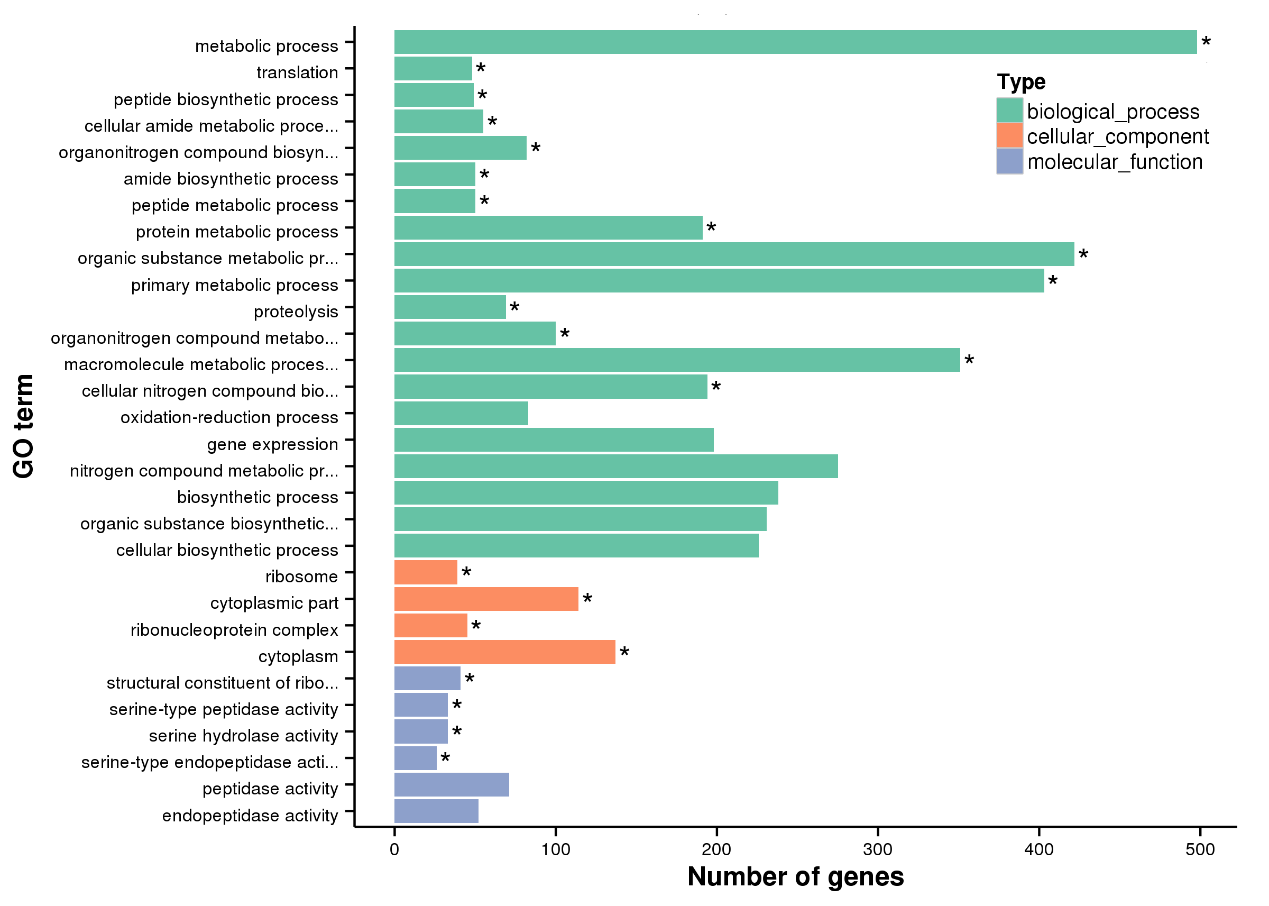


(B)


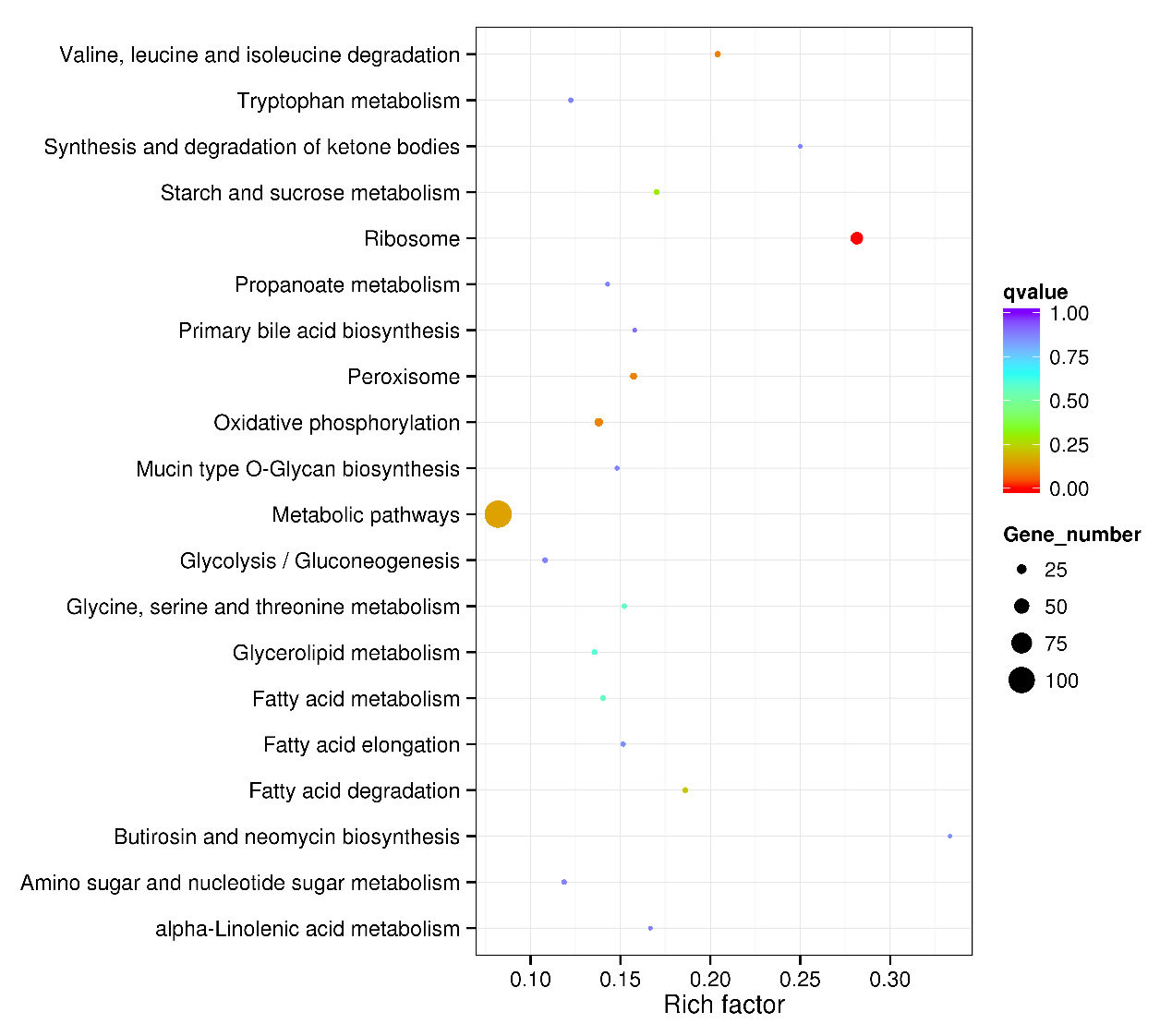


**Supplementary Fig. S3** **GO enrichment analysis (A) and statistics of KEGG pathway enrichment (B) for differentially expressed mRNA in lncRNA-mRNA co-expression analysis for D/E-1.46 vs D/E-2.75.** * denotes significant enrichment (*P*adj < 0.05, *P*adj is the adjusted *P*-value). The GO terms without full title: 1) Cellular amide metabolic process; 2) Organonitrogen compound biosynthetic process; 3) Organonitrogen compound metabolic process; 4) Cellular nitrogen compound biosynthetic process; 5) Organic substance metabolic process; 6) Nitrogen compound metabolic process; 7) Cellular nitrogen compound metabolic process; 8) Intracellular non-membrane-bounded organelle; 8) Structural constituent of ribosome. Rich factor is the ratio of number of differentially expressed genes in a certain pathway to number of all annotated genes in this pathway. qvalue is corrected *P* value by multiple hypothesis test. qvalue < 0.05 denotes significant differences.

(A)


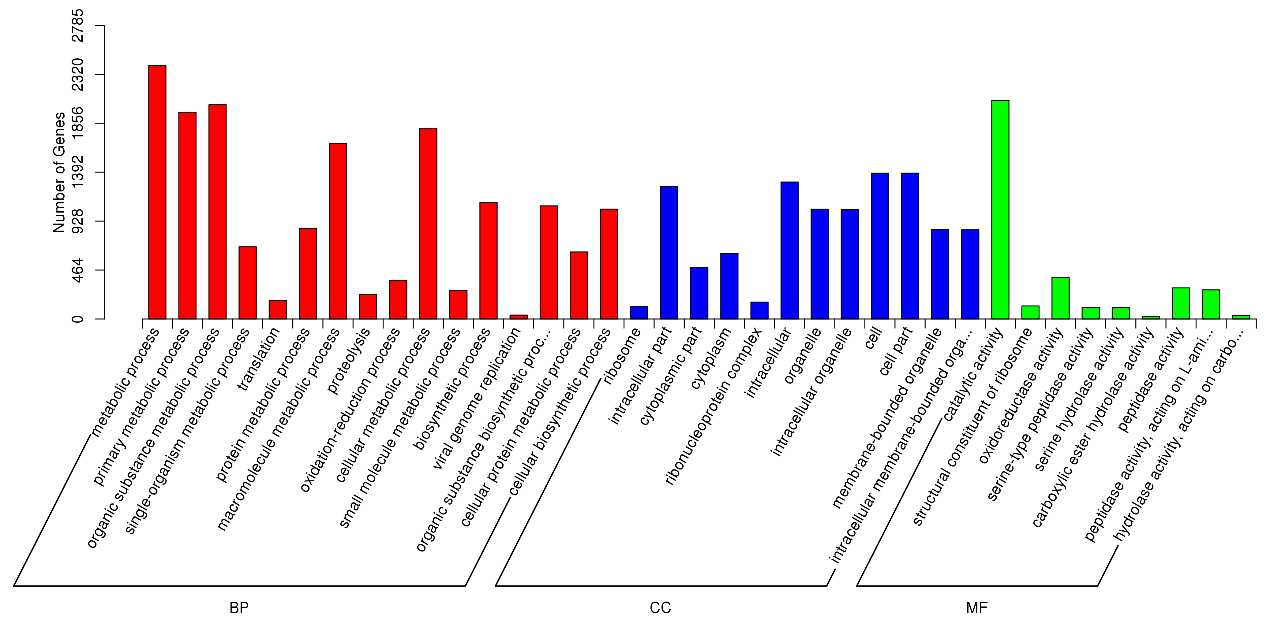


(B)


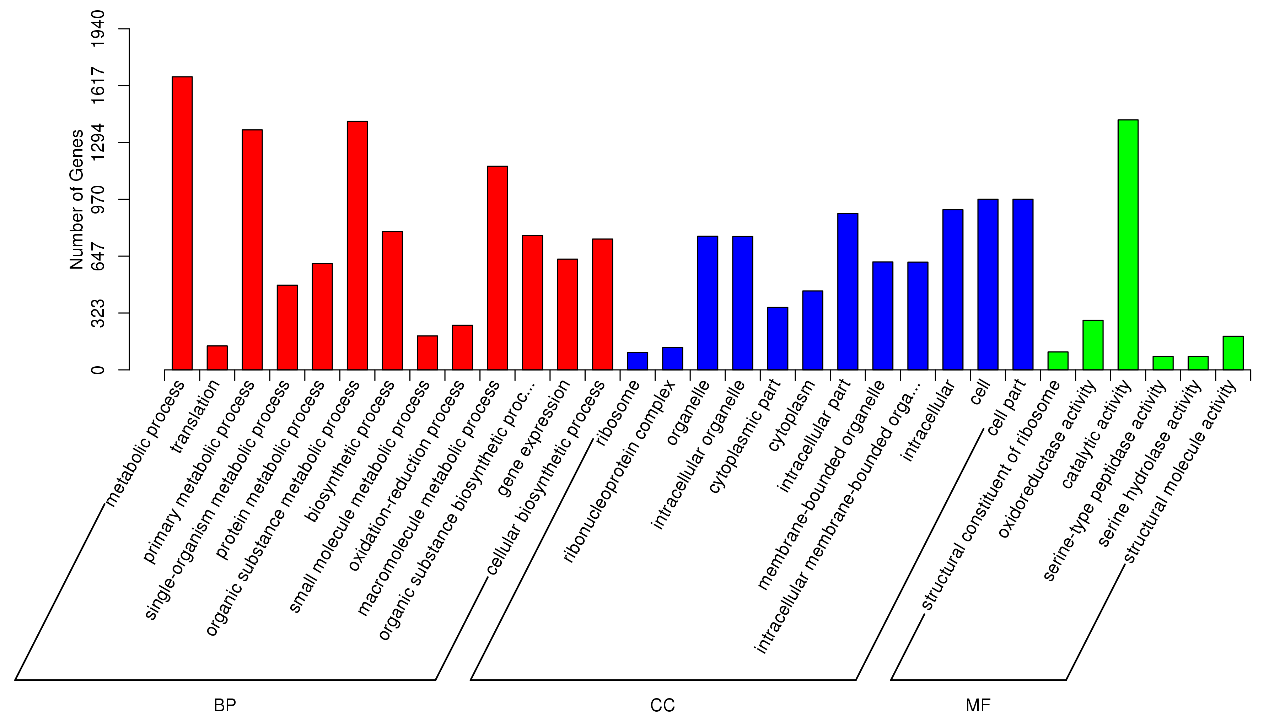


(C)


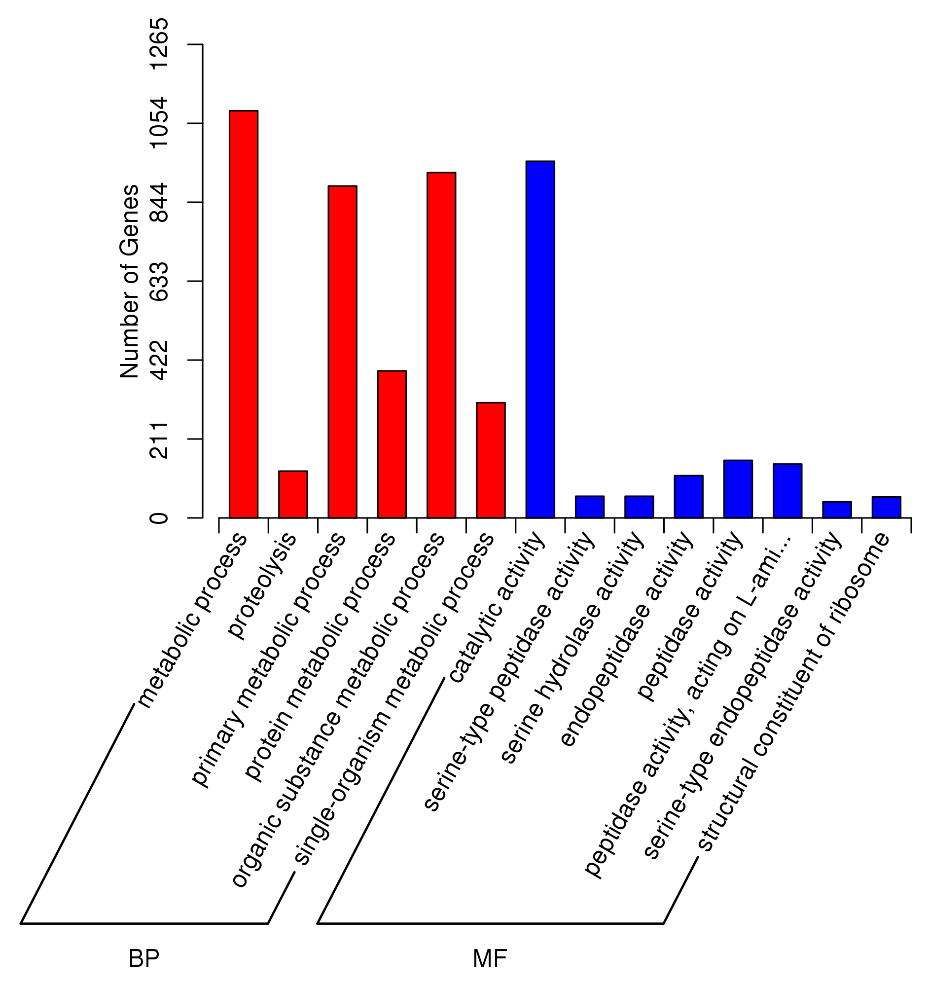


**Supplementary Fig. S4 GO enrichment analysis of differentially expressed mRNA.** BP: biological process, CC: cellular component, MF: molecular function. (A) D/E-1.46 vs D/E-0.61; (B) D/E-2.75 vs D/E-0.61; (C) D/E-1.46 vs D/E-2.75.

(A)


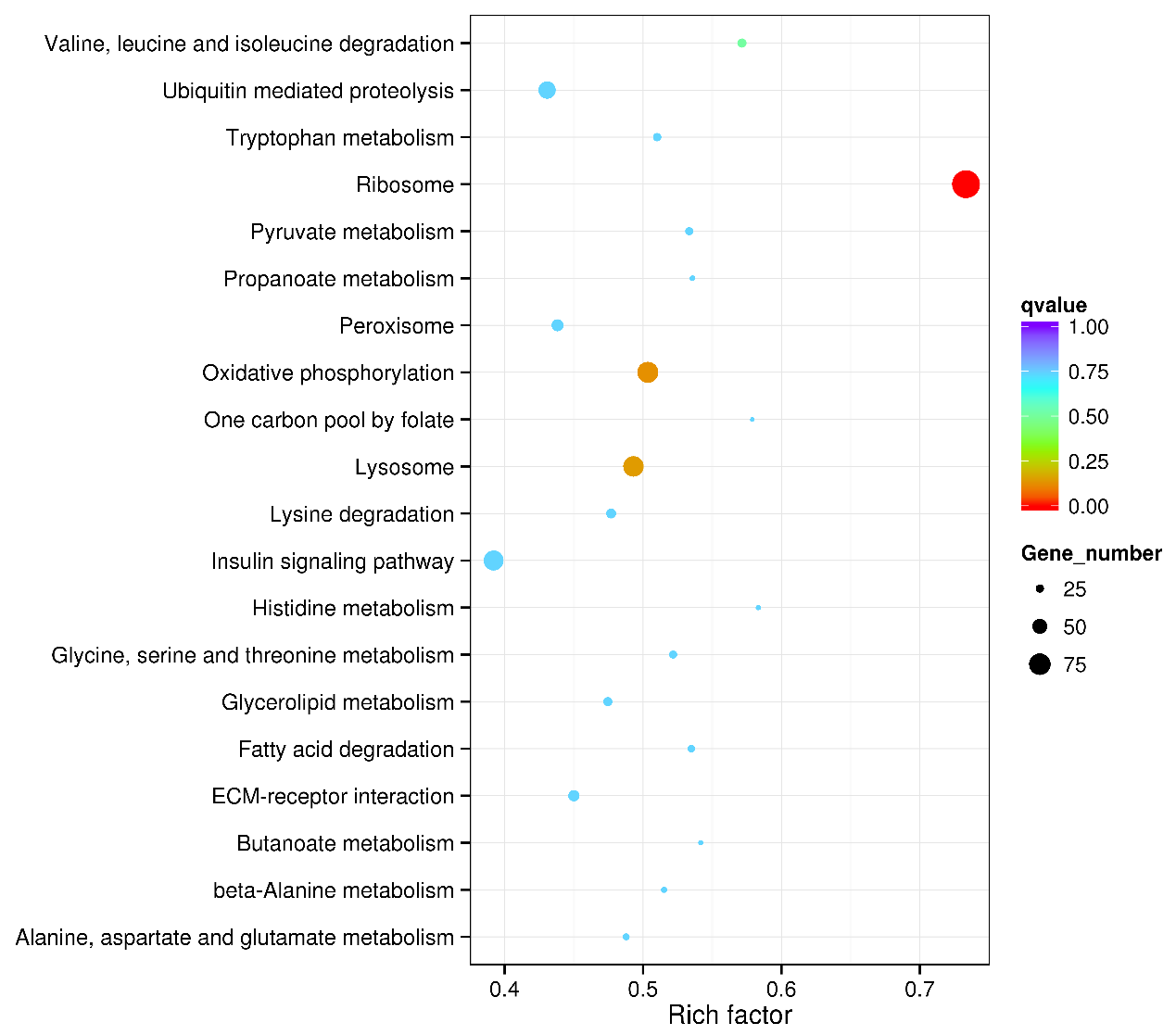


(B)


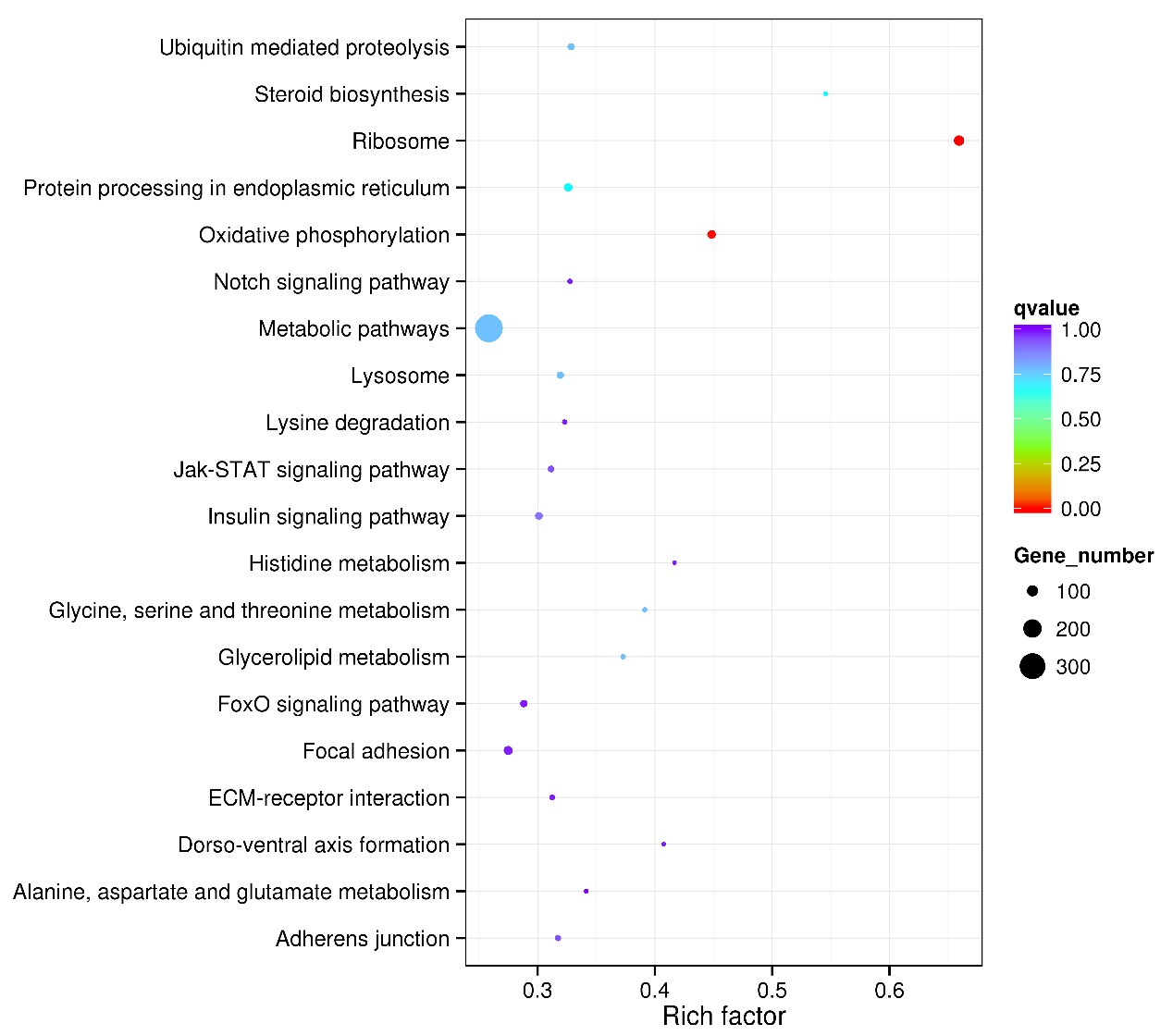


(C)

**
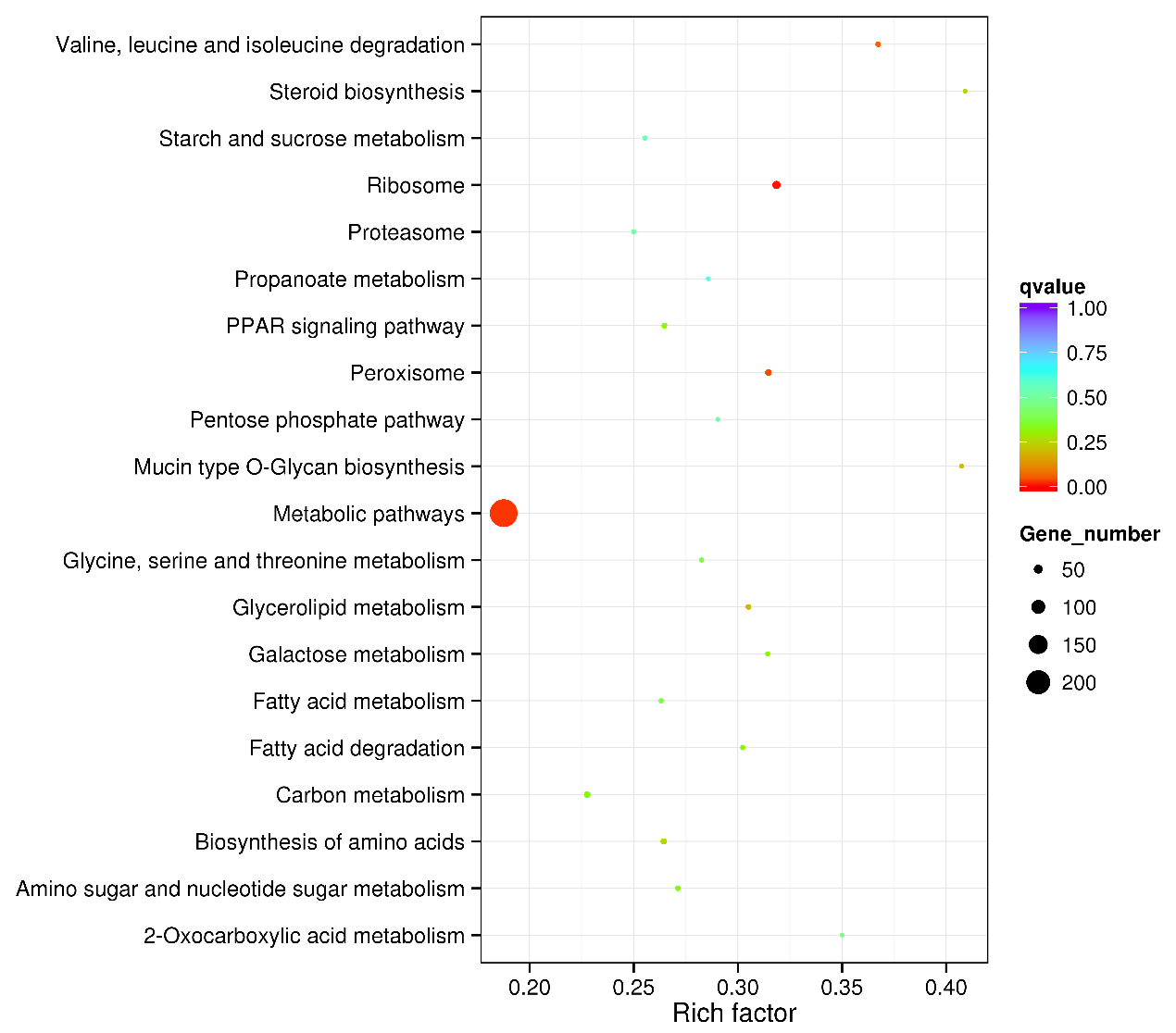
**

**Supplementary Fig. S5 KEGG classification of differentially expressed mRNA.** (A) D/E-1.46 vs D/E-0.61; (B) D/E-2.75 vs D/E-0.61; (C) D/E-1.46 vs D/E-2.75.

**(A)**


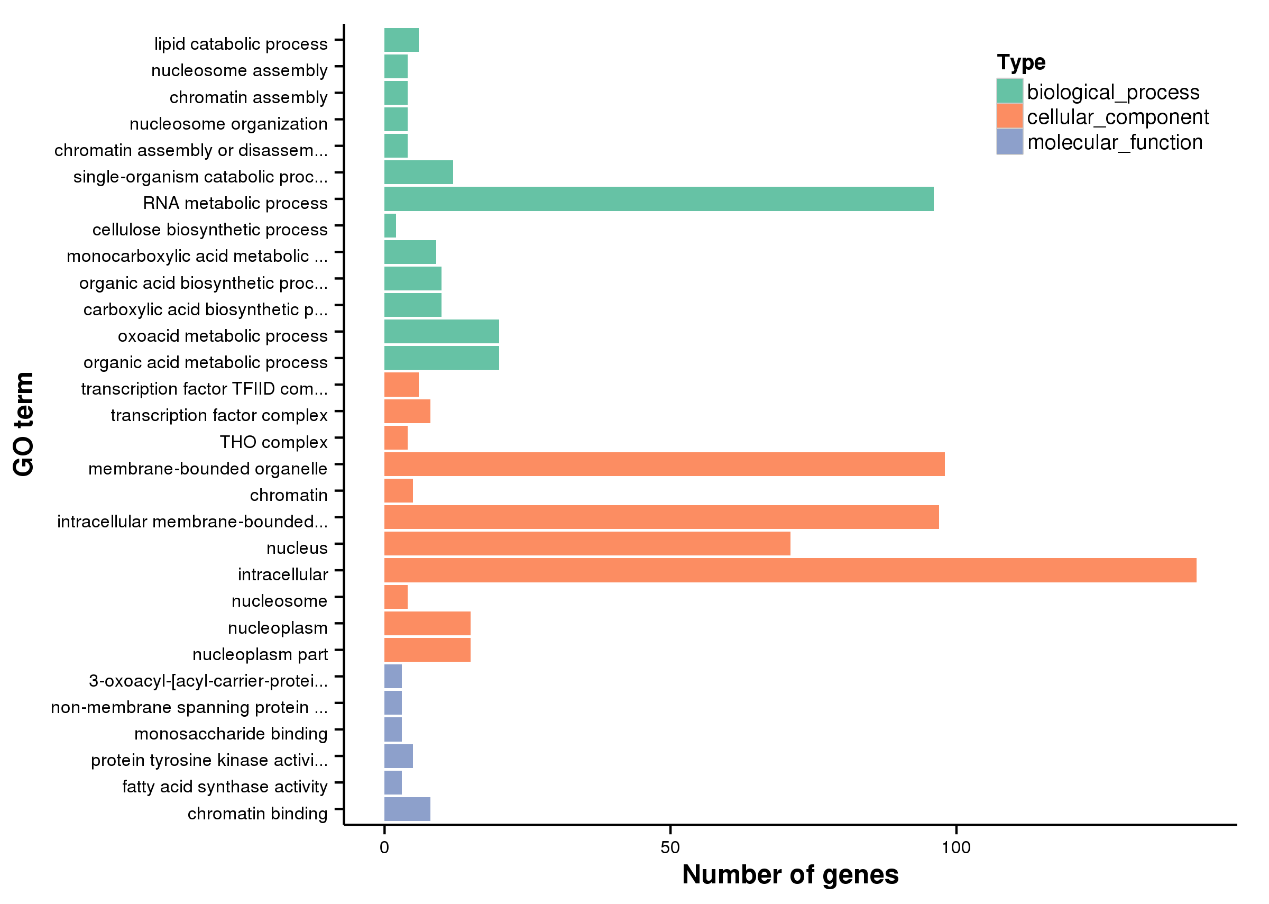


**(B)**


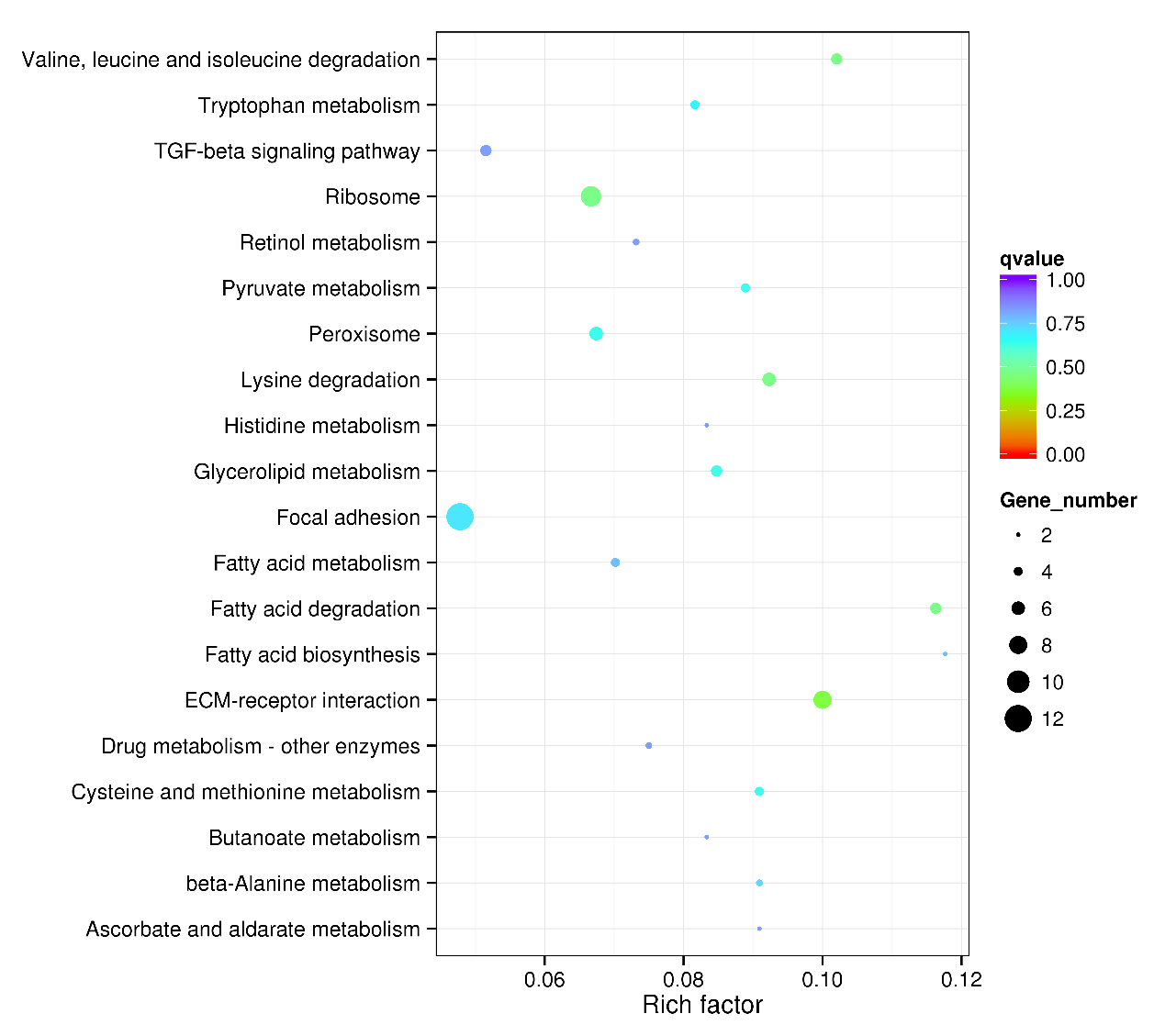


**Supplementary Fig. S6 GO enrichment analysis (A) and statistics of KEGG pathway enrichment (B) for differentially expressed mRNA in lncRNA-mRNA co-localization analysis for D/E-1.46 vs D/E-0.61.** * denotes significant enrichment (*P*adj < 0.05, *P*adj is the adjusted *P*-value). The GO terms without full title: 1) Chromatin assembly or disassembly; 2) Single-organism catabolic process; 3) Monocarboxylic acid metabolic process; 4) Organic acid biosynthetic process; 5) Carboxylic acid biosynthetic process; 6) Transcription factor TFIID complex; 7) Intracellular membrane-bounded organelle; 8) 3-oxoacyl-[acyl-carrier-protein] synthase activity; 9) Non-membrane spanning protein tyrosine kinase activity; 10) Protein tyrosine kinase activity. Rich factor is the ratio of number of differentially expressed genes in a certain pathway to number of all annotated genes in this pathway. qvalue is corrected *P* value by multiple hypothesis test. qvalue < 0.05 denotes significant differences.

(A)


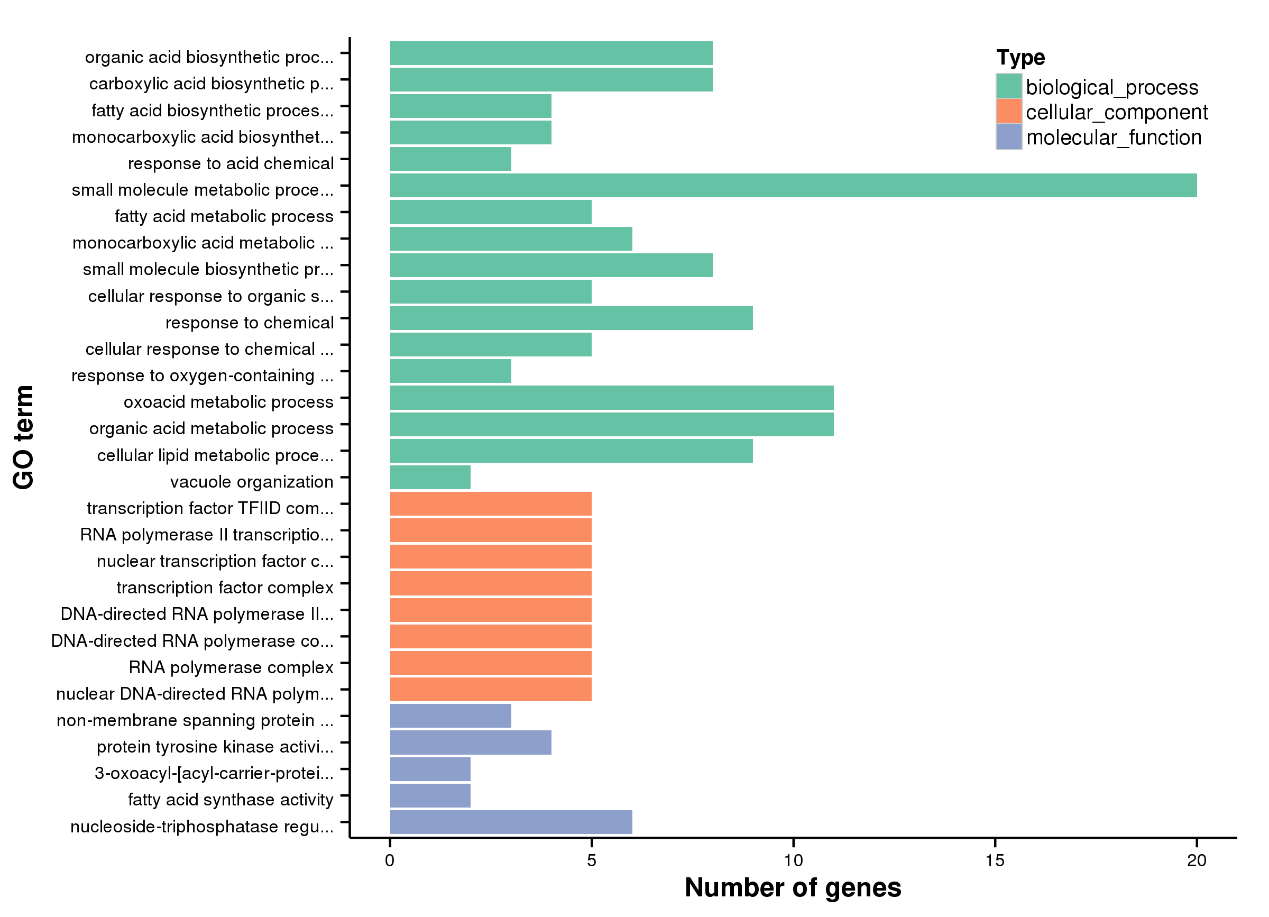


(B)


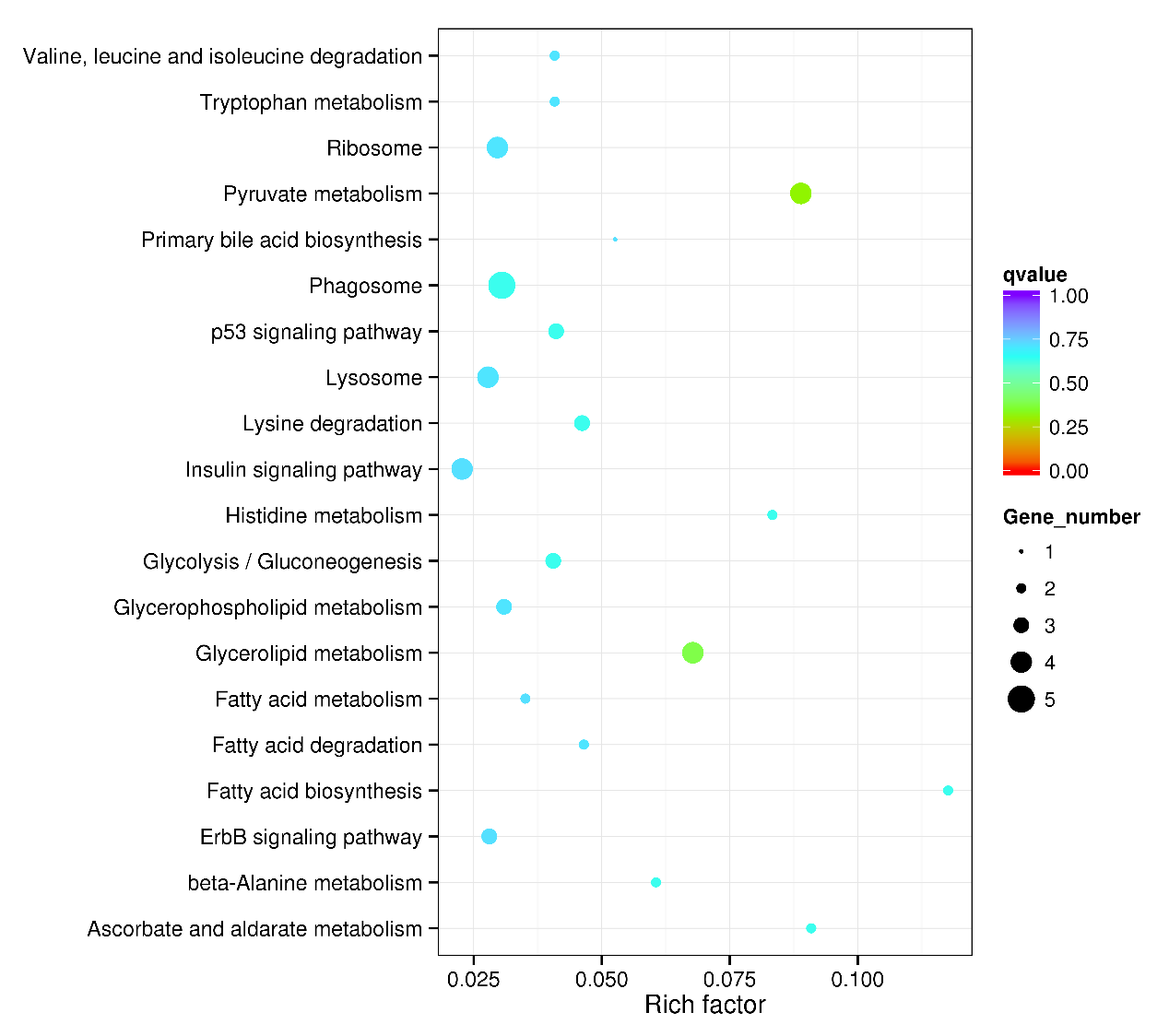


**Supplementary Fig. S7** **GO enrichment analysis (A) and statistics of KEGG pathway enrichment (B) for differentially expressed mRNA in lncRNA-mRNA co-localization analysis for D/E-2.75 vs D/E-0.61.** * denotes significant enrichment (*P*adj < 0.05, *P*adj is the adjusted *P*-value). The GO terms without full title: 1) Organic acid biosynthetic process; 2) Carboxylic acid biosynthetic process; 3) Fatty acid biosynthetic process; 4) Monocarboxylic acid biosynthetic process; 5) Small molecule biosynthetic process; 6) Cellular response to organic substance; 7) Cellular response to chemical stimulus; 8) Response to oxygen-containing compound; 9) Cellular lipid metabolic process; 10) Transcription factor TFIID complex; 11) DNA-directed RNA polymerase II, holoenzyme; 12) DNA-directed RNA polymerase complex; 13) Nuclear DNA-directed RNA polymerase complex; 14) Non-membrane spanning protein tyrosine kinase activity; 15) Protein tyrosine kinase activity; 16) 3-oxoacyl-[acyl-carrier-protein] synthase activity; 17) Nucleoside-triphosphatase regulator activity. Rich factor is the ratio of number of differentially expressed genes in a certain pathway to number of all annotated genes in this pathway. qvalue is corrected *P* value by multiple hypothesis test. qvalue < 0.05 denotes significant differences.

(A)


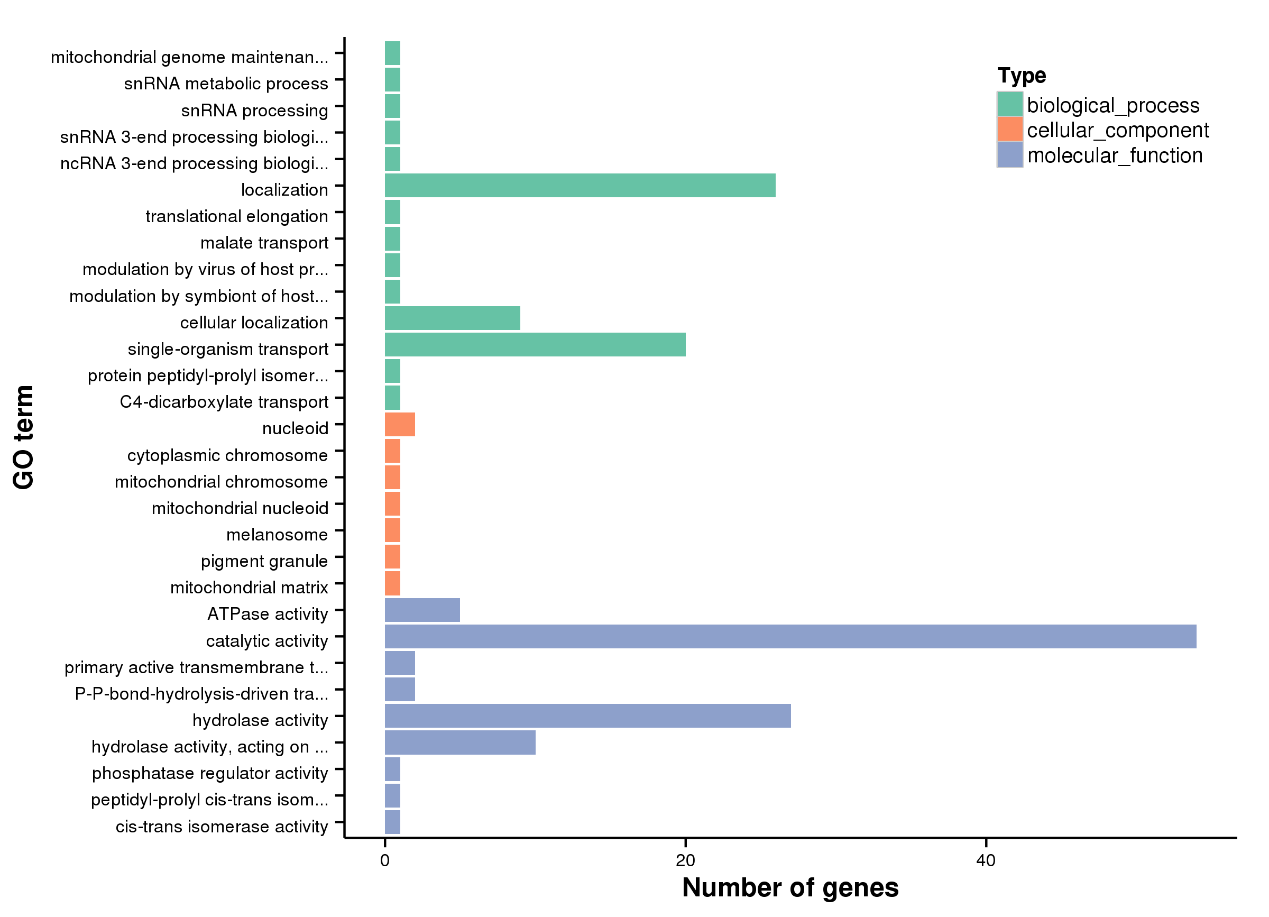


(B)


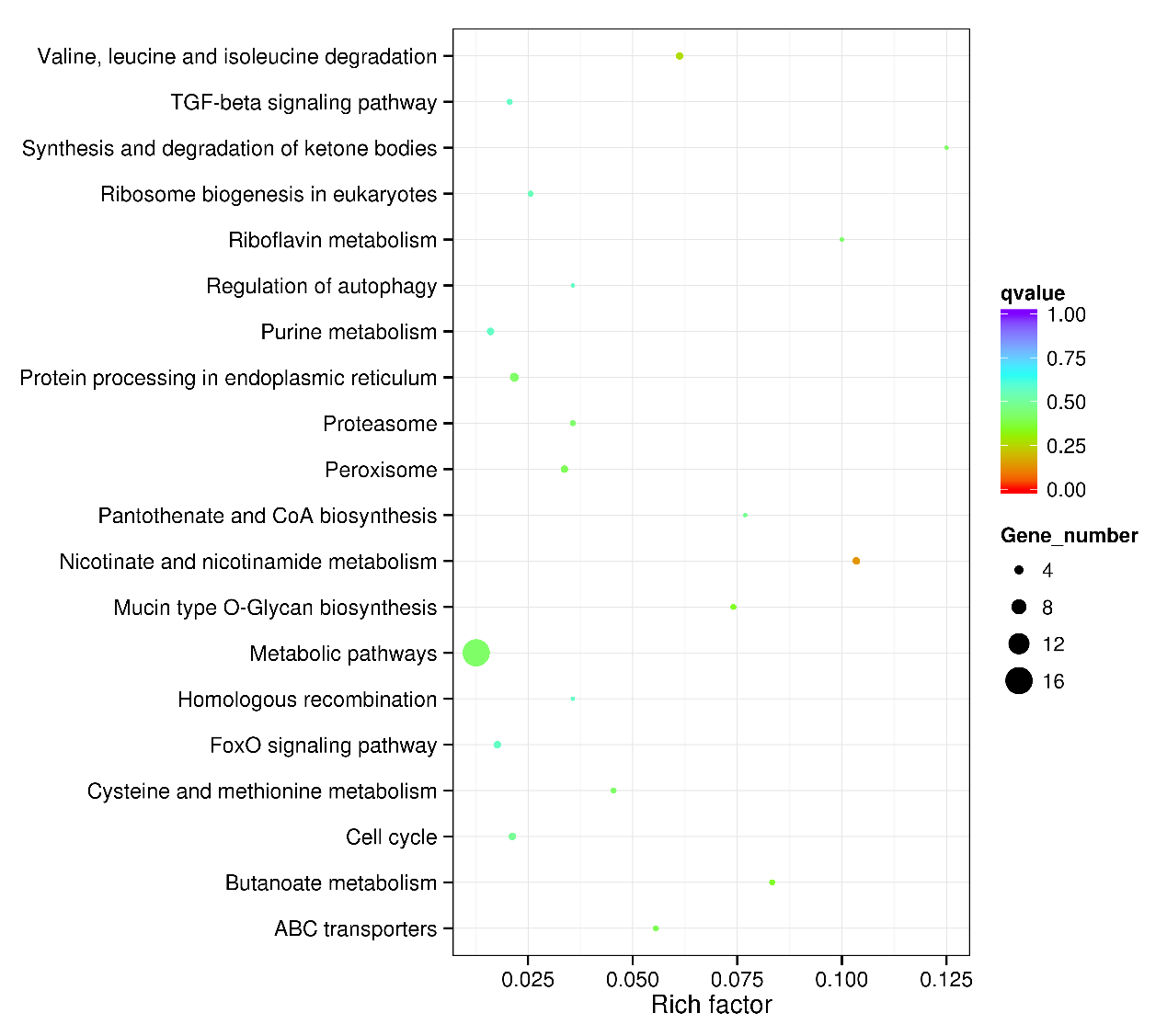


**Supplementary Fig. S8** **GO enrichment analysis (A) and statistics of KEGG pathway enrichment (B) for differentially expressed mRNA in lncRNA-mRNA co-localization analysis for D/E-1.46 vs D/E-2.75.** * denotes significant enrichment (*P*adj < 0.05, *P*adj is the adjusted *P*-value). The GO terms without full title: 1) Mitochondrial genome maintenance; 2) snRNA 3'-end processing biology; 3) ncRNA 3'-end processing biology; 4) Modulation by virus of host apoptotic process; 5) Modulation by symbiont of host apoptotic process; 6) Protein peptidyl-prolyl isomerization; 7) Primary active transmembrane transporter activity; 8) P-P-bond-hydrolysis-driven transmembrane transporter activity; 9) Hydrolase activity, acting on ester bonds; 10) Peptidyl-prolyl cis-trans isomerase activity. Rich factor is the ratio of number of differentially expressed genes in a certain pathway to number of all annotated genes in this pathway. qvalue is corrected *P* value by multiple hypothesis test. qvalue < 0.05 denotes significant differences.
